# Identification of a Human Trafficking Victim: A Simulation

**DOI:** 10.21980/J8293F

**Published:** 2024-07-31

**Authors:** Claire A Grosgogeat, Kelly Medwid, Rami H Mahmoud, Brooke Hensley

**Affiliations:** * University of Miami, Miller School of Medicine, Miami, FL; ^ Jackson Memorial Hospital/University of Miami, Department of Emergency Medicine, Miami, FL

## Abstract

**Audience:**

This case was designed for emergency medicine interns and residents.

**Introduction:**

Human trafficking is unfortunately an ever-growing and wide-reaching problem in the United States as well as the rest of the world. The International Labor Organization estimates 49.6 million people were affected by this modern-day slavery worldwide in 2021.^1^,^2^ The emergency department represents an opportunity to identify and provide aid to victims of human trafficking. Studies have shown that 63.3% of survivors interacted with the emergency department during their time of exploitation; however, most of these patients are not identified as human trafficking victims and opportunities for intervention are missed.^3^,^4^

**Educational Objectives:**

By the end of this simulation, participants will be able to: (1) Identify signs of human trafficking. (2) Demonstrate the ability to perform a primary and secondary assessment of a patient when there is concern for human trafficking. (3) Demonstrate the ability to appropriately separate an at-risk patient from a potential trafficker. (4) Identify resources and a reliable course of action to permanently remove the patient from the harmful situation.

**Educational Methods:**

A hybrid teaching model was employed that included both a lecture and a standardized patient simulation session followed by a structured debriefing session.

**Research Methods:**

A simulation with a standardized participant was implemented at an urban academic emergency department with a three-year EM residency program. Participants were evaluated with a survey prior to and after the simulation, where they responded to questions regarding human trafficking patients on a scale of 1 to 5, where 5 represented the greatest level of agreement. Nineteen emergency medicine interns and residents participated in this project.

**Results:**

Prior to simulation training, and after the lecture, residents were surveyed on their confidence in identifying and treating patients who are affected by trafficking, their level of previous training in this topic, and whether they considered trafficking an important issue in emergency medicine. When asked if human trafficking is an important issue faced by the emergency department, 15 of the 19 of residents who completed the survey rated the importance a 5/5 on a Likert scale ranging from 1-not important to 5. Residents were also asked if they had received prior training in human trafficking on a scale of never (1) to often (5). Eight residents responded with either never or close to never. Two months after the simulation, the residents were again sent an optional survey. Ten residents responded. All who participated in the simulation now rated themselves a 4/5 on a scale from not confident to very confident. Of those who did not attend the simulation, the median value was a 3/5. Out of the residents who attended the simulation training, every resident rated the experience 5 out of 5 in terms of usefulness. One hundred percent of residents would recommend simulation training on human trafficking to other emergency medicine residents.

**Discussion:**

This was an effective educational initiative because this education model allowed the residents to feel more comfortable identifying individuals affected by human trafficking, and all the residents who responded to the survey stated that they would recommend the use of simulation to others for education on human trafficking.

**Topics:**

High-fidelity simulation, human trafficking identification, human trafficking response.

## USER GUIDE

[Table t1-9-3-s1]

**Table t1-9-3-s1:** 

List of Resources:	
Abstract	1
User Guide	3
Instructor Materials	5
Pre-Simulation Presentation	5
Simulation Case	6
Operator Materials	18
Debriefing and Evaluation Pearls	22
Simulation Assessment	25

**Learner Audience:**

Interns, Junior Residents, Senior Residents

**Time Required for Implementation:**

**Instructor Preparation:** 2 hours including PowerPoint presentation

**Time for case:** 15–20 minutes for single cases

**Time for debriefing:** 10–30 minutes per case

**Recommended Number of Learners per Instructor:**

2 learners per 1 instructor

**Topics:**

High-fidelity simulation, human trafficking identification, human trafficking response.

**Objectives:**

By the end of this simulation, participants will be able to:

1. Identify signs of human trafficking.
2. Demonstrate the ability to perform a primary and secondary assessment of the patient when there is concern for human trafficking.
3. Demonstrate the ability to appropriately separate an at-risk patient from a potential trafficker.
4. Identify resources and a reliable course of action to permanently remove the patient from the harmful situation.

### Linked objectives and methods

Our goal for this project is to educate emergency medicine residents on how to identify as well as treat individuals affected by human trafficking. Our residents already participate in weekly didactic sessions and monthly simulation sessions as part of their emergency medicine education. For this curriculum, they were first given a lecture on how to identify victims of human trafficking, which focused on how to successfully question patients regarding human trafficking while providing a safe environment where the patient feels comfortable enough to discuss their situation. Next, our residents completed a survey to assess their baseline knowledge of human trafficking and their opinions on how important this topic is to their practice as emergency medicine physicians. Approximately one month later, they participated in a simulation where a standardized patient played the role of a human trafficking victim. Residents were not aware they would be participating in a simulation related to human trafficking prior to the simulation. The case involved a patient who is brought into the ED for left-sided chest pain accompanied by a person claiming to be his family member. The family member states that the patient fell down the stairs one week prior and has continued pain. The patient explains that he is an undocumented immigrant staying with this family member; however, he is evasive when answering questions, and the “family member” is overbearing. Our main objective is for the learner to pick up on this unusual dynamic and ask the patient**’**s “family member” to leave the room and question the patient by himself regarding the concern for trafficking. After the simulation, there is a 20-minute debriefing session that focuses on identifying and managing human trafficking victims in the emergency department. Two months after the simulation was completed, a survey was administered to all residents to reassess their knowledge and opinions regarding their role in identifying and treating victims of human trafficking. The residents who were unable to attend the simulation due to scheduling conflicts were the control.

### Associated content

A pre-simulation presentation (PowerPoint) and presenter notes for the presentation are included.

### Learner responsible content (optional)

Participants should attend the pre-simulation presentation.

### Results and tips for successful implementation

Prior to simulation training, and after the lecture, residents were surveyed on their confidence in identifying and treating patients who are affected by trafficking, their level of previous training in this topic, and whether they considered trafficking an important issue in emergency medicine. When asked if human trafficking is an important issue faced by the emergency department, 15 of the 19 residents who completed the survey rated the importance a 5/5 on a Likert scale ranging from 1-not important to 5-essential with one person and three people rating an importance of 3/5 or 4/5, respectively. Residents were also asked if they had received prior training in human trafficking on a scale of never (1) to often (5). Eight residents responded with either never or close to never, four residents responded neutral, six responded a 4/5, and one resident responded often. As a follow-up to this, the type of training/education received was surveyed, with 78.9% answering “lecture.” The categories “Documents,” “Small Group Discussions,” and “None,” all received 15.8% of votes, and notably, “Simulation” received 0%.

Two months after the simulation, the residents were again sent an optional survey. Ten residents responded. Four participated in the simulation; six did not. Residents who participated in simulation training felt more confident in their ability to identify signs of human trafficking victims and in their ability to intervene. All who participated in the simulation rated themselves now a 4/5 on a scale from not confident to very confident. Of those who did not attend the simulation, the median value was 3/5. Out of the residents who attended the simulation training, every resident rated the experience 5 out of 5 in terms of usefulness. One hundred percent of residents would recommend simulation training on human trafficking to other emergency medicine residents.

In both the pre- and post-simulation surveys, 100% of residents believed they would encounter victims of human trafficking in their current or future practice. One surprising answer was that when asked if residents believed they had enough resources to effectively intervene if encountering a victim of human trafficking, 75% of residents who participated in the simulation answered “maybe.” We believe that this answer was regarding their new knowledge that many human trafficking victims go through the healthcare system undetected, and we hope that this awareness will make these physicians more proactive in identifying those who are affected.

## Supplementary Information





## Figures and Tables

**Figure f1-9-3-s1:**
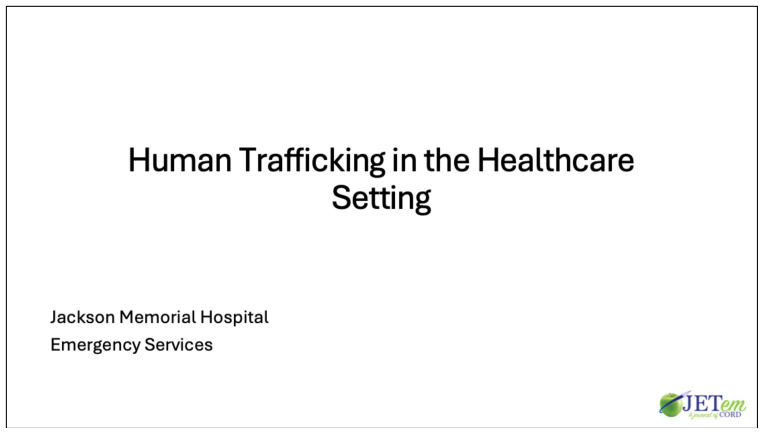
Please see associated PowerPoint file

**Table t2-9-3-s1:** 

#1	Complete Blood Count
#2	Complete Metabolic Panel
#3	Coagulation Studies
#4	Chest X-Ray
#5	eFAST
#6	Pelvis X-Ray

**Table t3-9-3-s1:** 

**Vitals:**	Heart Rate (HR) 110	Blood Pressure (BP) 134/82
	Respiratory Rate (RR) 22	Temperature (T) 97.6°F (36.6°C)
	Oxygen Saturation (O_2_Sat) 98% on room air	

**Table t4-9-3-s1:** 

*Complete blood count (CBC)*	
White blood count (WBC)	13.7 × 1000/mm^3^
Hemoglobin (Hgb)	12.2 g/dL
Hematocrit (HCT)	36.3%
Platelet (Plt)	257 × 1000/mm^3^

**Table t5-9-3-s1:** 

*Complete metabolic panel (CMP)*	
Sodium	135 mEq/L
Potassium	4.6 mEq/L
Chloride	105 mEq/L
Bicarbonate (HCO_3_)	23 mEq/L
Blood Urea Nitrogen (BUN)	11 mg/dL
Creatinine (Cr)	0.06 mg/dL
Glucose	93 mg/dL
Total bilirubin	1.1 mg/dL
Aspartate aminotransferase (AST)	54 units/L
Alanine aminotransferase (ALT)	43 units/L

**Table t6-9-3-s1:** 

*Coagulation Studies*	
Prothrombin time	10.0 seconds
Partial thromboplastin time	31.5 seconds
International normalized ratio	0.9

**Figure f2-9-3-s1:**
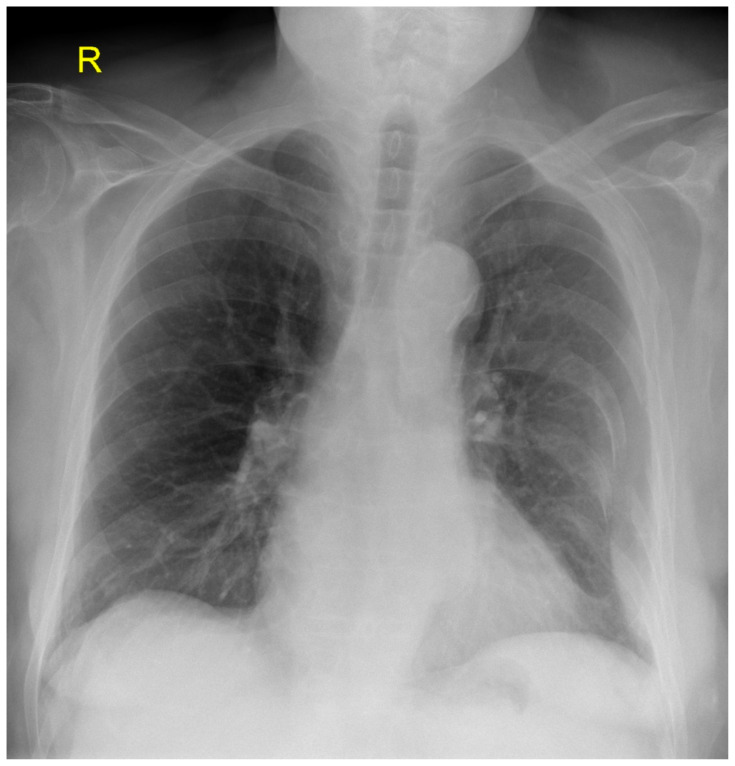
Left-sided 3rd-7th rib fractures with 6th and 8th rib fractures displaced. No pneumothorax or pleural fluid collection.

**Figure f3-9-3-s1:**
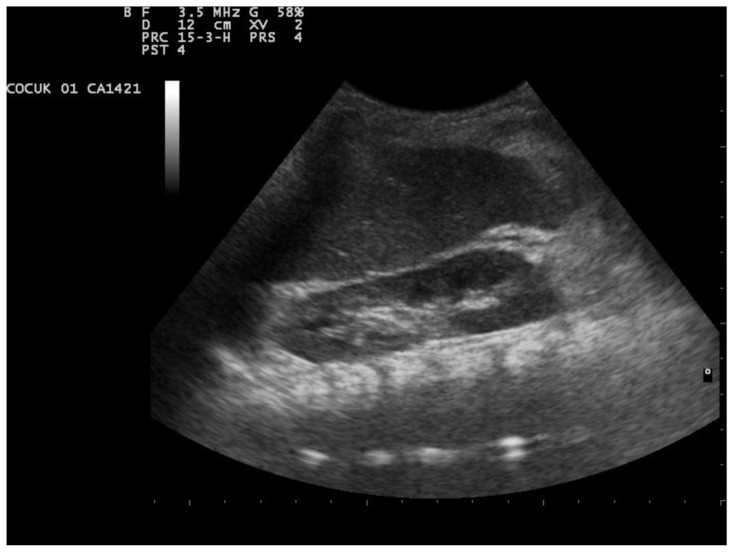


**Figure f4-9-3-s1:**
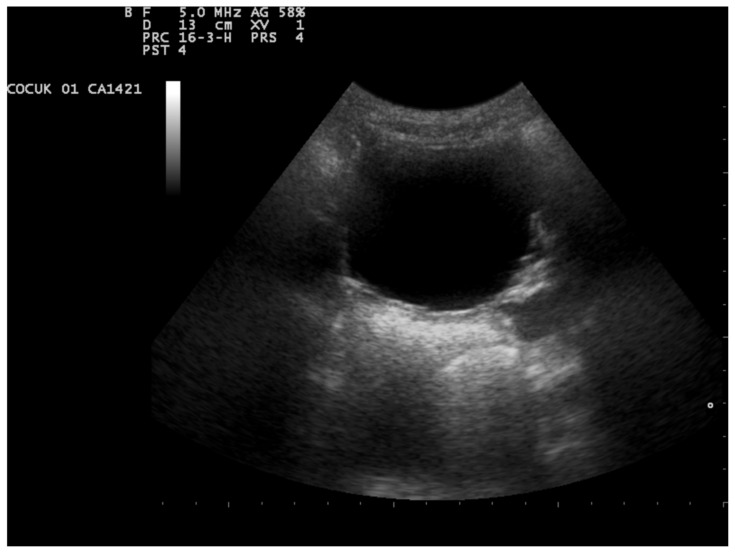


**Figure f5-9-3-s1:**
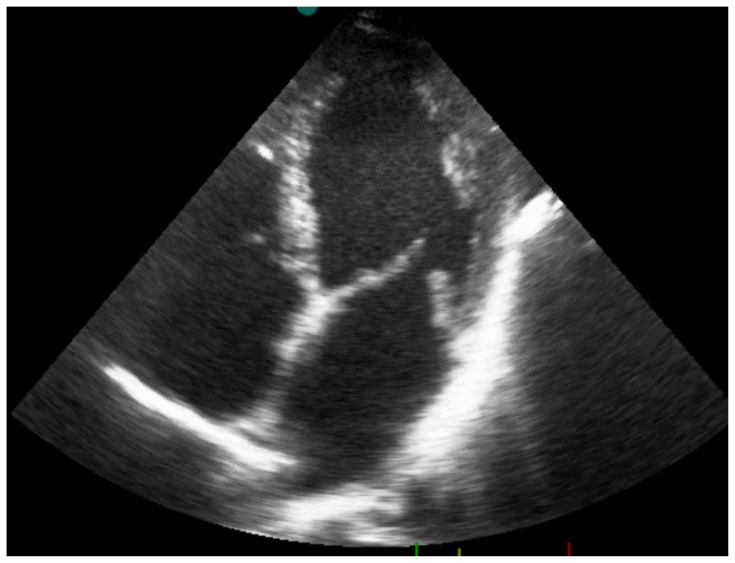


**Figure f6-9-3-s1:**
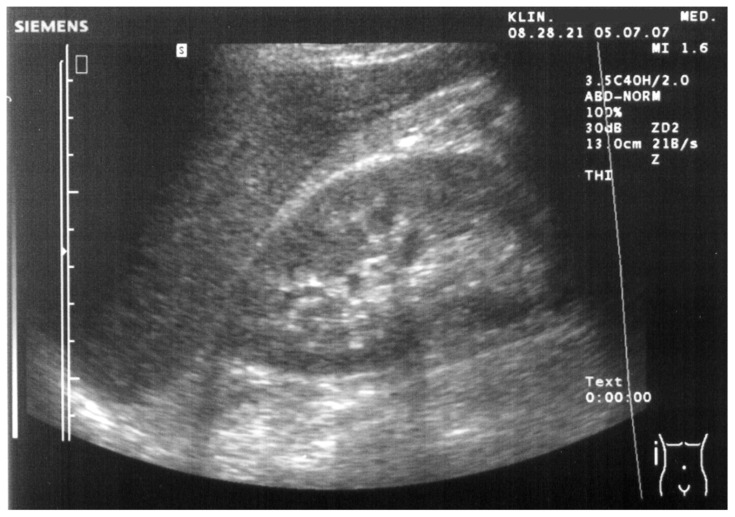


**Figure f7-9-3-s1:**
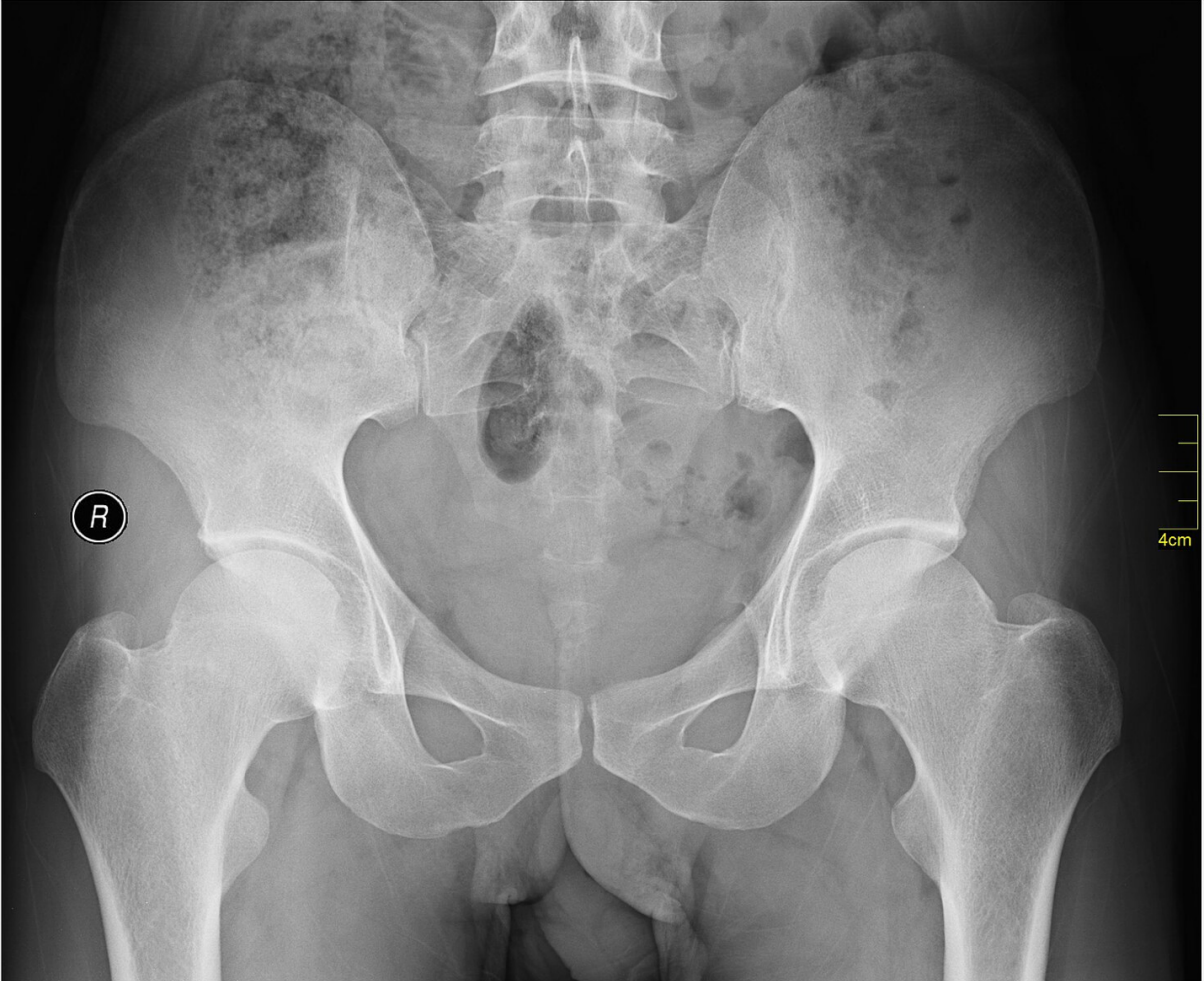


**Table t7-9-3-s1:** SIMULATION EVENTS TABLE:

Minute (state)	Participant action/ trigger	Patient status (simulator response) & operator prompts	Monitor display (vital signs)
0:00 (Base-line)	Team Roles Identified. Airway, breath sounds, and circulation assessed.	The patient appears anxious and is withdrawn.	T 97.9° F HR 110 BP 134/82 RR 22 O2 98%
2 mins	History and physical exam completed. Areas of ecchymoses in various stages of healing found throughout extremities and back. Pain control (Tylenol or an NSAID) is ordered for the patient. Anticipated management mistake: team will not notice signs of human trafficking including ecchymoses in various stages of healing.	Family member controls the conversation, answering most questions. Patient continues to be anxious and withdrawn.	
3 mins	Suspicious behavior is noted between the patient and the “family member.” The team asks to hear more from the patient instead of the family member and they ask that the family member step out of the room. Participants will come up with a plausible reason for the family member to leave the room. Anticipated management mistakes: Family member is not asked to leave the room. A plausible reason for the family member to leave the room is not given (ie, exam must be done in private, paperwork for the family member to complete outside, etc).	Family member leaves the room. Patient appears more at ease. Anticipated management mistake: Family member stays in the room and continues to control the narrative. Participants will be unable to obtain an accurate history as long as family member is present. If a reason is not given for the family member to leave the room, family member will object once, and then the family member will leave the room.	
6 mins	Trauma-informed care used to interview the patient to determine if patient is at risk of human trafficking. Anticipated management mistakes: Trauma-informed care is not used to interview the patient AND/OR family member is still in the room.	Patient responds positively to trauma informed care. The heart rate of the patient will also now normalize and was due to initial anxiety about the situation. Anticipated management mistake: Patient remains reserved and engages minimally in conversation if trauma informed care is not used. If family member is still in the room, family member will become upset and tell the patient to leave. The patient will leave against medical advice unless the participant actively de-escalates the encounter and separates the patient and family member.	T 97.9° F HR 86 BP 128/84 RR 18 O2 97%
8 mins	Participant orders chest x-ray. FAST exam and pelvic XR available upon request but not necessary. eFAST recommended.	Chest X-ray results return. Acute left-sided 3rd-7th rib fractures with 6th and 8th rib fractures displaced. No pneumothorax.	
10 mins (Case Completion)	Patient given resources on human trafficking or agrees to be removed from the situation. Anticipated management mistake: Failure to provide concrete resources on human trafficking and a reliable course of action to be removed from the situation.	Patient accepts resources and agrees to be removed from the situation. Anticipated management mistake: If no resources are presented, patient will not agree to be removed from the situation.	

**Table t8-9-3-s1:** 

**Critical Actions:** Team roles identified. Airway, breath sounds, and circulation are assessed. A focused history and physical are taken. CXR and or rib X-ray is ordered. The team notes a suspicious dynamic between the patient and his “family member.” The team ask the family member to leave the room. Trauma-informed care is used to interview the patient regarding a concern for human trafficking. The patient is given resources regarding human trafficking and how to get help.	0:00

**Table t9-9-3-s1:** 

	Milestone	Did not achieve level 1	Level 1	Level 2	Level 3
1	**Emergency Stabilization (PC1)**	□ Did not achieve Level 1	□ Recognizes abnormal vital signs	□ Recognizes an unstable patient, requiring intervention Performs primary assessment Discerns data to formulate a diagnostic impression/plan	□ Manages and prioritizes critical actions in a critically ill patient Reassesses after implementing a stabilizing intervention
2	**Performance of focused history and physical (PC2)**	□ Did not achieve Level 1	□ Performs a reliable, comprehensive history and physical exam	□ Performs and communicates a focused history and physical exam based on chief complaint and urgent issues	□ Prioritizes essential components of history and physical exam given dynamic circumstances
3	**Diagnostic studies (PC3)**	□ Did not achieve Level 1	□ Determines the necessity of diagnostic studies	□ Orders appropriate diagnostic studies. Performs appropriate bedside diagnostic studies/procedures	□ Prioritizes essential testing Interprets results of diagnostic studies Reviews risks, benefits, contraindications, and alternatives to a diagnostic study or procedure
4	**Diagnosis (PC4)**	□ Did not achieve Level 1	□ Considers a list of potential diagnoses	□ Considers an appropriate list of potential diagnosis May or may not make correct diagnosis	□ Makes the appropriate diagnosis Considers other potential diagnoses, avoiding premature closure
5	**Pharmacotherapy (PC5)**	□ Did not achieve Level 1	□ Asks patient for drug allergies	□ Selects an medication for therapeutic intervention, consider potential adverse effects	□ Selects the most appropriate medication and understands mechanism of action, effect, and potential side effects Considers and recognizes drug-drug interactions
6	**Observation and reassessment (PC6)**	□ Did not achieve Level 1	□ Reevaluates patient at least one time during case	□ Reevaluates patient after most therapeutic interventions	□ Consistently evaluates the effectiveness of therapies at appropriate intervals
7	**Disposition (PC7)**	□ Did not achieve Level 1	□ Appropriately selects whether to admit or discharge the patient	□ Appropriately selects whether to admit or discharge Involves the expertise of some of the appropriate specialists	□ Educates the patient appropriately about their disposition Assigns patient to an appropriate level of care (ICU/Tele/Floor) Involves expertise of all appropriate specialists
9	**General Approach to Procedures (PC9)**	□ Did not achieve Level 1	□ Identifies pertinent anatomy and physiology for a procedure Uses appropriate Universal Precautions	□ Obtains informed consent Knows indications, contraindications, anatomic landmarks, equipment, anesthetic and procedural technique, and potential complications for common ED procedures	□ Determines a back-up strategy if initial attempts are unsuccessful Correctly interprets results of diagnostic procedure
20	**Professional Values (PROF1)**	□ Did not achieve Level 1	□ Demonstrates caring, honest behavior	□ Exhibits compassion, respect, sensitivity and responsiveness	□ Develops alternative care plans when patients’ personal beliefs and decisions preclude standard care
22	**Patient centered communication (ICS1)**	□ Did not achieve level 1	□ Establishes rapport and demonstrates empathy to patient (and family) Listens effectively	□ Elicits patient’s reason for seeking health care	□ Manages patient expectations in a manner that minimizes potential for stress, conflict, and misunderstanding. Effectively communicates with vulnerable populations, (at risk patients and families)
23	**Team management (ICS2)**	□ Did not achieve level 1	□ Recognizes other members of the patient care team during case (nurse, techs)	□ Communicates pertinent information to other healthcare colleagues	□ Communicates a clear, succinct, and appropriate handoff with specialists and other colleagues Communicates effectively with ancillary staff

## INSTRUCTOR MATERIALS

Identification of a Human Trafficking Victim: Pre- Simulation Presentation[Fig f1-9-3-s1]

**Case Title:** Identification of a Human Trafficking Victim: A Simulation

**Case Description & Diagnosis (short synopsis):** Mr. Smith is a 22-year-old male who is brought into the emergency department for left-sided chest pain. He is accompanied by a person who is claiming to be his family member. He states that he fell down the stairs one week ago resulting in left-sided chest pain that has not improved. The team should go into the room to take a history and physical and recognize that something about the dynamic between the patient and the “family member” is off. The patient is a victim of human trafficking. The “family member” is really his employer. The patient will be evasive when answering questions, and the family member will be very overbearing. The learner should perform a focused history and physical and pick up on this abnormal behavior. At that time, the learner should ask the patient’s family member to leave the room and question the patient by himself regarding the concern for trafficking.

**Equipment or Props Needed:** Moulage for the standardized patient includes bruising to the left chest that is “acute,” (with purple/red make-up) and other random bruises that appear more chronic (using yellow-brown).

**Actors needed:** A family member role: (1) Answer the majority of the questions regarding the fall during the history. (2) If they try to separate the family member from the patient, resist unless they present a valid reason. A nurse: place the patient on the monitor. An X-ray technician: obtain the X-rays.

**Stimulus Inventory:** [Table t2-9-3-s1]

**Background and brief information:** The patient is a 22-year-old male complaining of chest pain after a fall one week ago and brought into the emergency department by his “family member.” A male patient with a female family member was selected to demonstrate how human trafficking victims and offenders can be of any gender; therefore, any gender may be used for each role.

**Initial presentation:** The patient presents with left-sided chest pain after a fall one week ago.

**How the scene unfolds:** As the encounter begins, the female family member controls the dialogue and patient narrative, answering most of the questions addressed to the patient. The patient appears uncomfortable with the situation. The participant may note other red flags of human trafficking including delayed access to care and evidence of trauma in varying stages of healing. If the patient is separated from the family member, the patient responds well to empathetic comments and will provide insight into his situation, including physical abuse and that he is a victim of human trafficking in the form of labor trafficking. The participant can then initiate a conversation regarding options to get the patient to a safe situation and other resources.

- Ideal Scenario Flow
  - The patient appears anxious and withdrawn. He is guarding his left chest with a family member in the room
  - Physician: history, physical exam
  - Nursing/Tech
    - Place the patient on a monitor
- Anticipated Management Mistakes
  - Failure to recognize signs of human trafficking and separate the potential victim from the potential human trafficker
  - Failure to provide concrete resources on human trafficking and a reliable course of action to be removed from the situation
  - Failure to note ecchymoses in various stages of healing throughout the body on physical exam

**Critical actions:**

1. Team roles identified.
2. Airway, breath sounds, and circulation are assessed.
3. A focused history and physical are taken.
4. CXR and or rib X-ray is ordered.
5. The team notes a suspicious dynamic between the patient and his “family member.”
6. The team ask the family member to leave the room.
7. Trauma-informed care is used to interview the patient regarding a concern for human trafficking.
8. The patient is given resources regarding human trafficking and how to get help.

**Case Title:** Identification of a Human Trafficking Victim: A Simulation

**Chief Complaint:** Left-sided chest pain since a fall one week ago.[Table t3-9-3-s1]

**General Appearance:** Cooperative, anxious, flat affect.

**Primary Survey:**

- **Airway:** Intact
- **Breathing:** Intact
- **Circulation:** Intact

**History:**

- **History of present illness:** The patient reportedly fell one week ago on the stairs. He states that he slipped and landed on his left side. He has a constant, 8/10, sharp pain that radiates across his left chest. It is aggravated by movement, talking, and deep breaths. It is alleviated by Tylenol. He has had multiple prior visits to the ED for anxiety and depression.
- **Past medical history:** Anxiety, depression
- **Past surgical history:** None.
- **Medications:** Tylenol for pain, Sertraline (Zoloft) for depression and anxiety
- **Allergies:** None.
- **Social history:** He is originally from Poland. He works at the family member’s restaurant. He lives with the family member. He drinks alcohol socially, denies tobacco and drug use.
- **Family history:** Non-contributory.

**Secondary Survey/Physical Examination:**

- **General appearance:** Awake and alert. Appears withdrawn and is hesitant to answer questions.
- **HEENT:** Pupils equal, reactive, PERRLA, no hemotympanum, no oropharyngeal bleeding
  ○ **Head:** within normal limits (wnl)
  ○ **Eyes:** wnl
  ○ **Ears:** wnl
  ○ **Nose:** wnl
  ○ **Throat:** wnl
- **Neck:** Trachea midline
- **Heart:** Sinus tachycardia, no murmurs
- **Lungs:** Symmetric expansion, diminished breath sounds on left side
- **Abdominal/GI:** Normal active bowel sounds, no bruits, soft, non-tender without guarding or rebound, liver and spleen not palpable, no hepatosplenomegaly
- **Genitourinary:** No bleeding, no wounds
- **Rectal:** wnl
- **Extremities:** Ecchymoses in various stages of healing on the extremities.
- **Back:** wnl
- **Neuro:** Glasgow Coma Scale (GCS) 15
- **Skin:** Warm, dry, capillary refill 2 seconds. Large ecchymosis on the left lateral chest. Ecchymoses in various stages of healing on the chest as well as the extremities and face.
- **Lymph:** wnl
- **Psych:** Mildly agitated, anxious, flattened affect.

[Table t4-9-3-s1] [Table t5-9-3-s1] [Table t6-9-3-s1]

*Chest X-Ray*

Knipe H, Left-sided rib fractures. Case study, Radiopaedia.org (Accessed on 22 Feb 2024)

https://doi.org/10.53347/rID-31240. CC BY-NC-SA 3.0 DEED[Fig f2-9-3-s1]

*eFAST:*

Dilmen, N. Ultrasound images of spleen. WikiMedia.

https://commons.wikimedia.org/wiki/File:Ultrasound_Scan_ND_110225135932_1402260.jpg. 2012. CC-BY-SA-3.0[Fig f3-9-3-s1]

Dilmen, N. Medical ultrasound image. WikiMedia.

https://commons.wikimedia.org/wiki/File:Ultrasound_Scan_ND_0105095458_1005300.png. 2011. CC-BY-SA-3.0,2.5,2.0,1.0[Fig f4-9-3-s1]

Ciernik, M. ProapsZCMiCh. WikiMedia.

https://commons.wikimedia.org/wiki/File:ProapsZCMiCh_.gif. 2017. CA-BY-SA-4.0[Fig f5-9-3-s1]

Ultrasound-image of Morison’s Pouch. WikiMedia.

https://commons.wikimedia.org/wiki/File:MorisonNoText.png. 2007. CA-BY-SA-2.5-2.0-1.0[Fig f6-9-3-s1]

*Pelvic X-Ray*

Dilmen, N. Medical X-rays. WikiMedia. https://commons.wikimedia.org/wiki/File:Medical_XRay_imaging_EXY04_nevit.jpg. 2011. CC-BY-SA-3.0[Fig f7-9-3-s1]

## OPERATOR MATERIALS

[Table t7-9-3-s1]

**Diagnosis:**

The patient is identified as a victim of human trafficking. He also has acute left-sided 3rd–7th rib fractures with 6th and 8th rib fractures displaced.

**Disposition:**

The patient accepts resources to be removed from the situation of human trafficking.

## DEBRIEFING AND EVALUATION PEARLS

### Identification of a Human Trafficking Victim

Human trafficking involves the use of force, fraud, or coercion to obtain some type of labor or commercial sex act. It occurs in all ages, races, genders and nationalities.^1^

The traffickers will often prey on people with some vulnerability. This may include:

- A psychological or emotional issue
- Some sort of financial hardship
- The lack of a social safety net
- Some sort of natural disaster
- Undocumented immigrants
- People from an area with political instability^1^

Human trafficking is unfortunately an ever-growing and wide-reaching problem. The International Labor Organization estimates 49.6 million people were affected by this modern-day slavery worldwide in 2021, and the number may even be higher due to those who are not identified.^1^,^2^

One place we might be able to identify these people is in the emergency department. Studies have shown that 63.3% of survivors interacted with the emergency department during their time of exploitation; however, most of these people are not identified as human trafficking victims and are unfortunately sent back into their situation.^3^,^4^

**Other debriefing points:**

As with every patient, first make sure that your patient is stable and assess their airway, breathing, and circulation.

Make sure you perform a complete history and physical, with an interpreter as necessary, who is not the person who accompanies the patient.

When performing a history, check for the following red flags regarding human trafficking:

- A delay in presentation for medical care.
- A discrepancy between the history given and the clinical presentation or pattern of injury.
- Frequent untreated injuries or infections.
- A scripted, memorized, mechanically recited, or restricted history.
- A reluctance to speak in the presence of the accompanying individual.
- An overly attentive or hyper-vigilant companion who is reluctant to leave the patient and insists on interpreting or speaking for the patient.
- A patient who is hyper-vigilant, fearful, untrusting, or lacks eye contact.
- A person who does not own/have control of his/her documents or money.

If the patient is accompanied by someone, find a way to interview the patient alone.

Ways to get the patient alone may include:

- Asking the other person to step out.
- Saying that it is hospital policy that you interview adults privately.
- Ask the patient questions as you privately take them for various studies.
- Ask the family member to step aside to ask them questions as another member of your team privately interviews the patient.

Screening Questions to be asked to the patient alone:

- Are you able to come and go as you please?
- Where do you sleep and eat?
- Does someone hold your identity or immigration documents?
- What type of work do you do?
- Have you ever been forced to exchange sex for food or shelter?
- Have you been asked to have sex with multiple partners?
- Do you have to meet a quota of money before you go home?
- Have threats from your employer made you fearful of leaving your job?

If you suspect that trafficking might be present:

- Alert your attending and charge nurse along with social worker.
- Place the patient in a room alone.
- Educate the patient on their rights and resources.

Realize that you cannot force help and assistance on a patient. If he or she refuses help, you must respect the patient’s wishes.

However, you can provide them with the Human Trafficking hotline, 1-888-373 -7888, and encourage them to return to the ED at any time.

Mandatory Reporting is required, however, for minors and vulnerable adults.

If they do accept help, contact your social worker, and call the national human trafficking hotline, 1-888-373 -7888, for resources in your area along with local law enforcement

## SIMULATION ASSESSMENT

*Identification of a Human Trafficking Victim: A Simulation*

Learner: _________________________________________

***Assessment Timeline***

This timeline is to help observers assess their learners. It allows observer to make notes on when learners performed various tasks, which can help guide debriefing discussion.[Table t8-9-3-s1]

**Critical Actions:**

□ Team roles identified.
□ Airway, breath sounds, and circulation are assessed.
□ A focused history and physical are taken.
□ CXR and or rib X-ray is ordered.
□ The team notes a suspicious dynamic between the patient and his “family member.”
□ The team ask the family member to leave the room.
□ Trauma-informed care is used to interview the patient regarding a concern for human trafficking.
□ The patient is given resources regarding human trafficking and how to get help.

**Summative and formative comments:**

**Milestones assessment:** [Table t9-9-3-s1]
