# Supplementary material for: Identification of a Human Trafficking Victim: A Simulation
Source: J Educ Teach Emerg Med. 2024 Jul 31;9(3):S1–S29. doi: 10.21980/J8293F (PMC11312875; doi:10.21980/J8293F)
Supplement: Supplementary file 1 [file 9-3-S1-Supp1.pptx]

## Slide 1
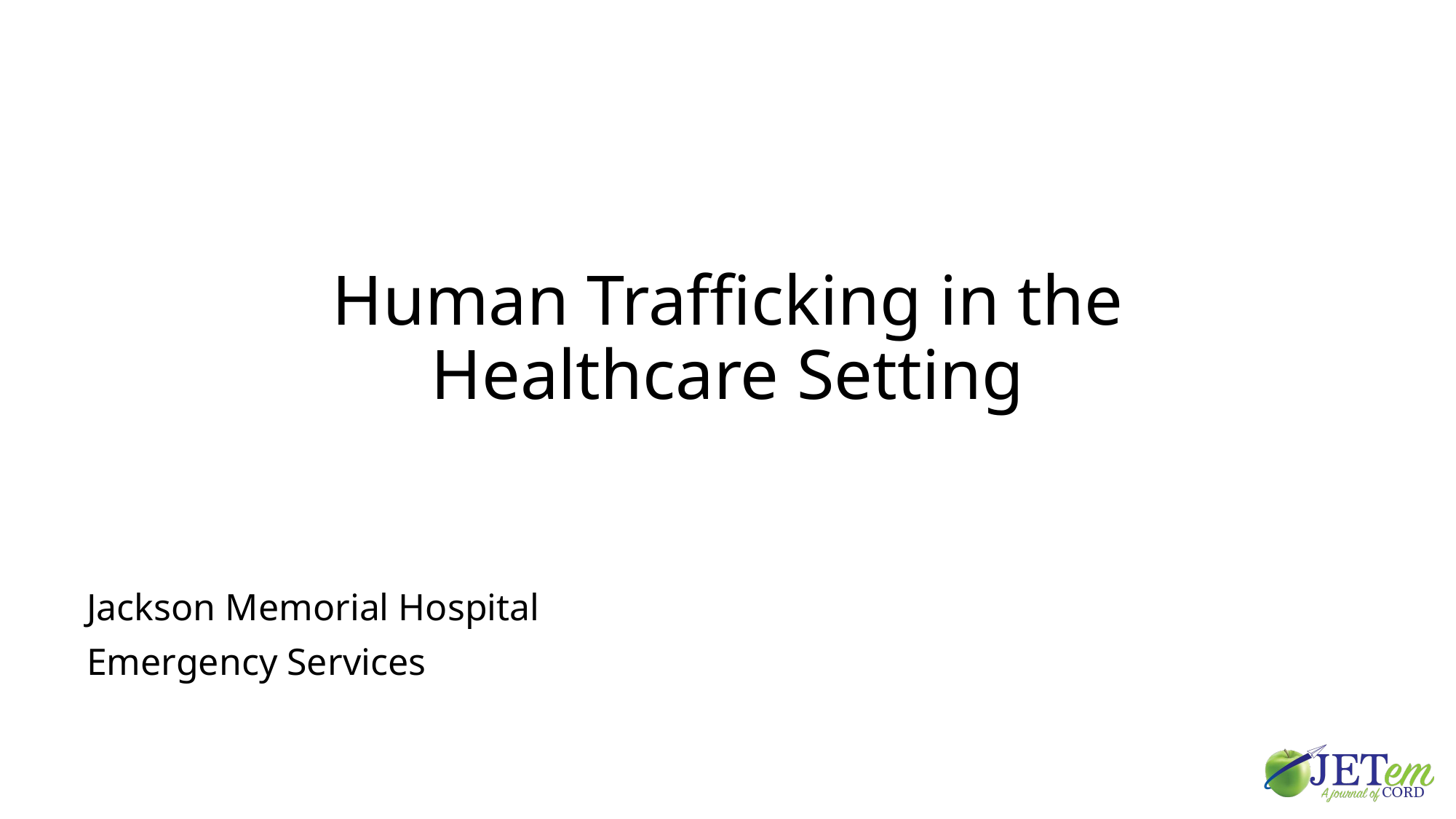

# Human Trafficking in the Healthcare Setting
Jackson Memorial Hospital
Emergency Services

## Slide 2
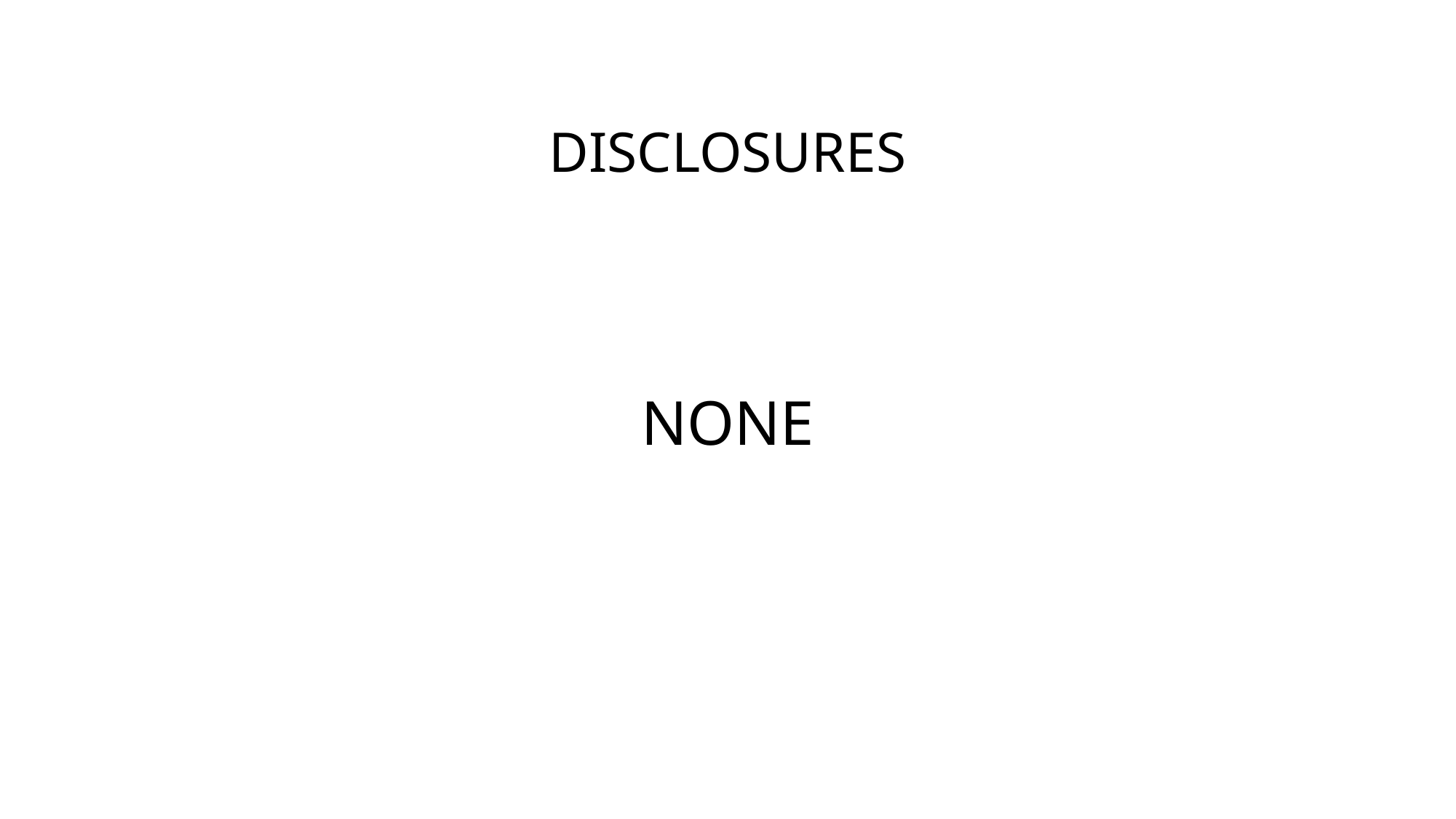

# DISCLOSURES
NONE

## Slide 3
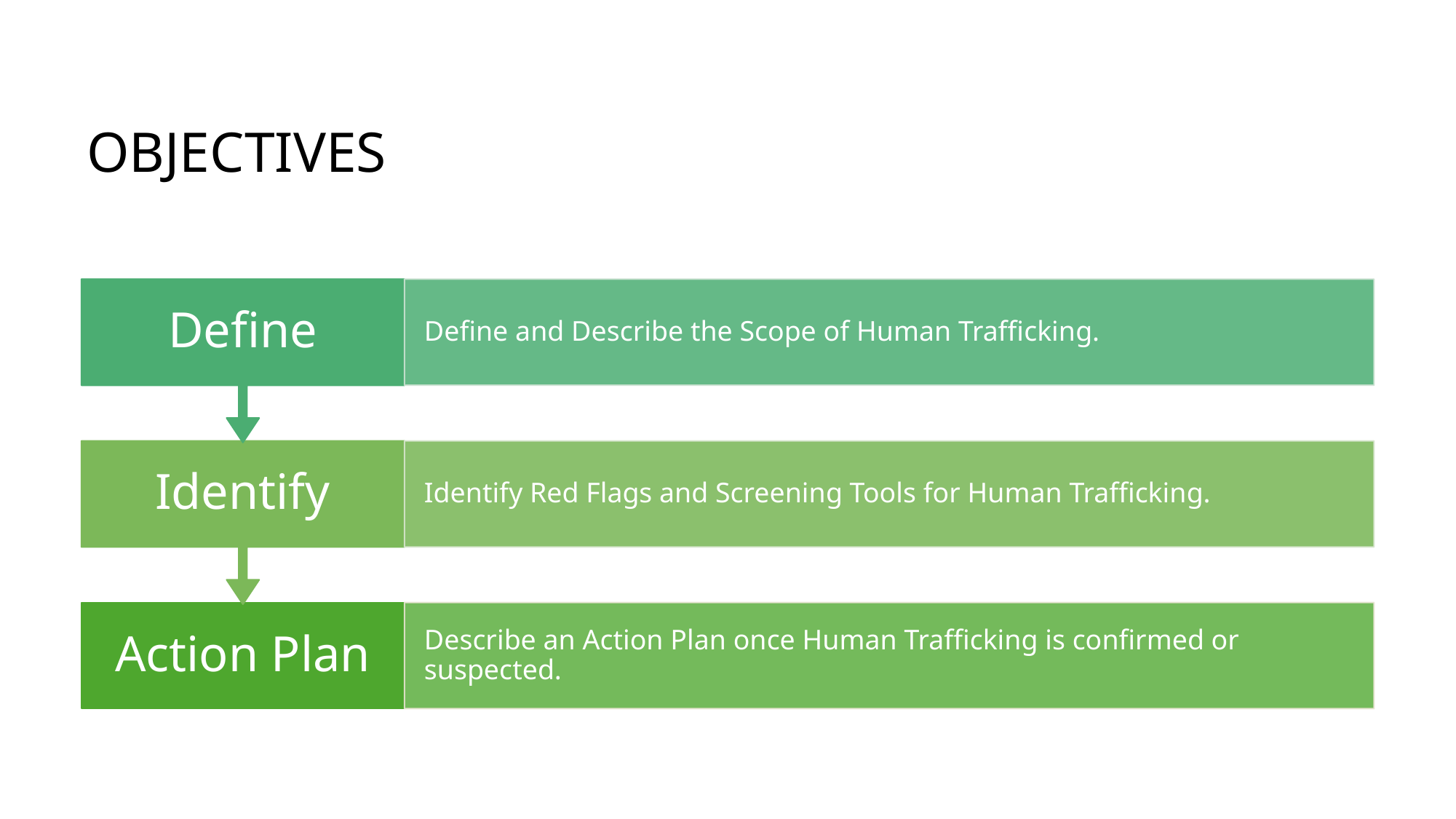

# OBJECTIVES
Define
Define and Describe the Scope of Human Trafficking.
Identify
Identify Red Flags and Screening Tools for Human Trafficking.
Action Plan
Describe an Action Plan once Human Trafficking is confirmed or suspected.

## Slide 4
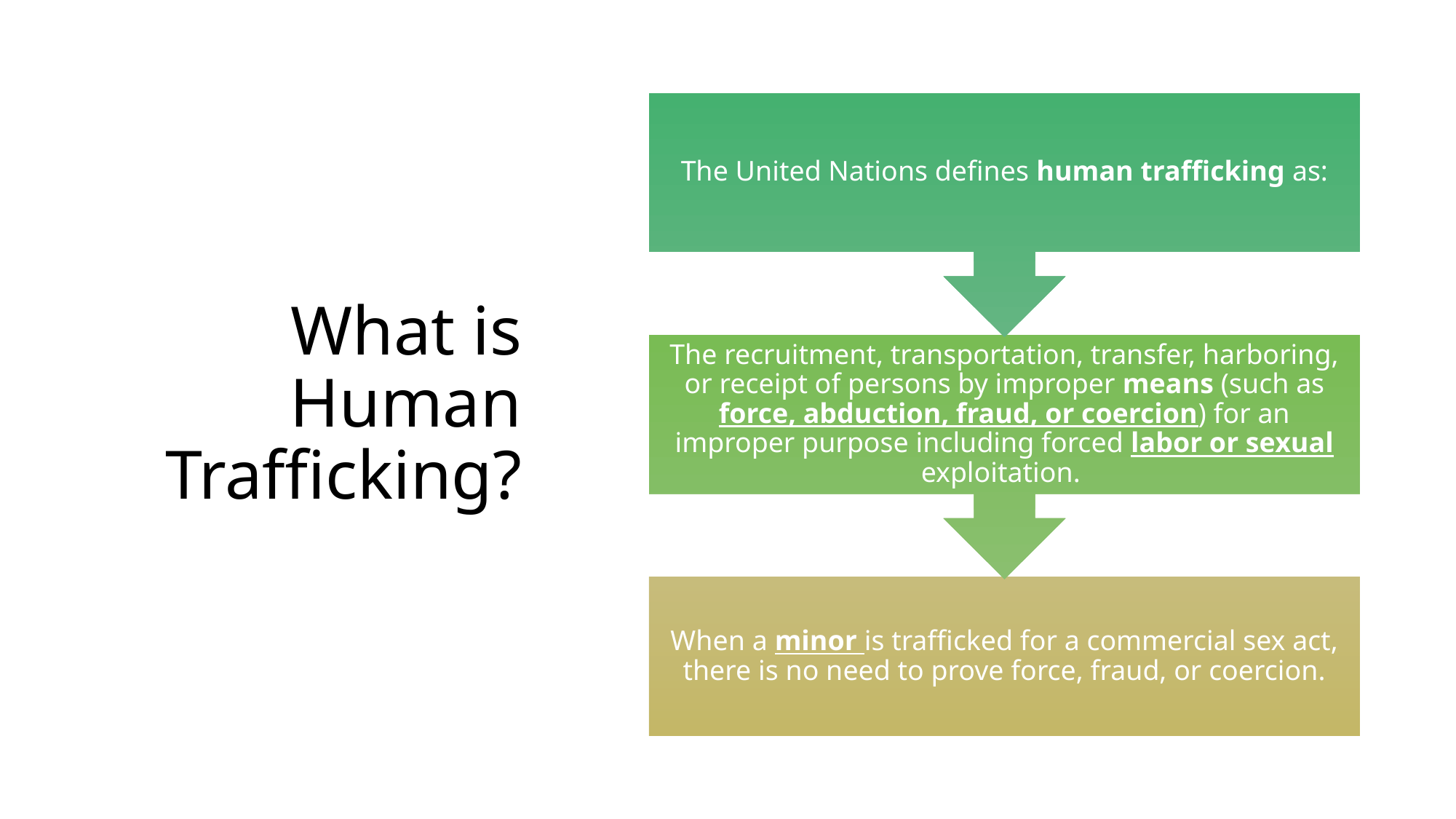

The United Nations defines human trafficking as:
The recruitment, transportation, transfer, harboring, or receipt of persons by improper means (such as force, abduction, fraud, or coercion) for an improper purpose including forced labor or sexual exploitation.
When a minor is trafficked for a commercial sex act, there is no need to prove force, fraud, or coercion.
# What is Human Trafficking?

## Slide 5
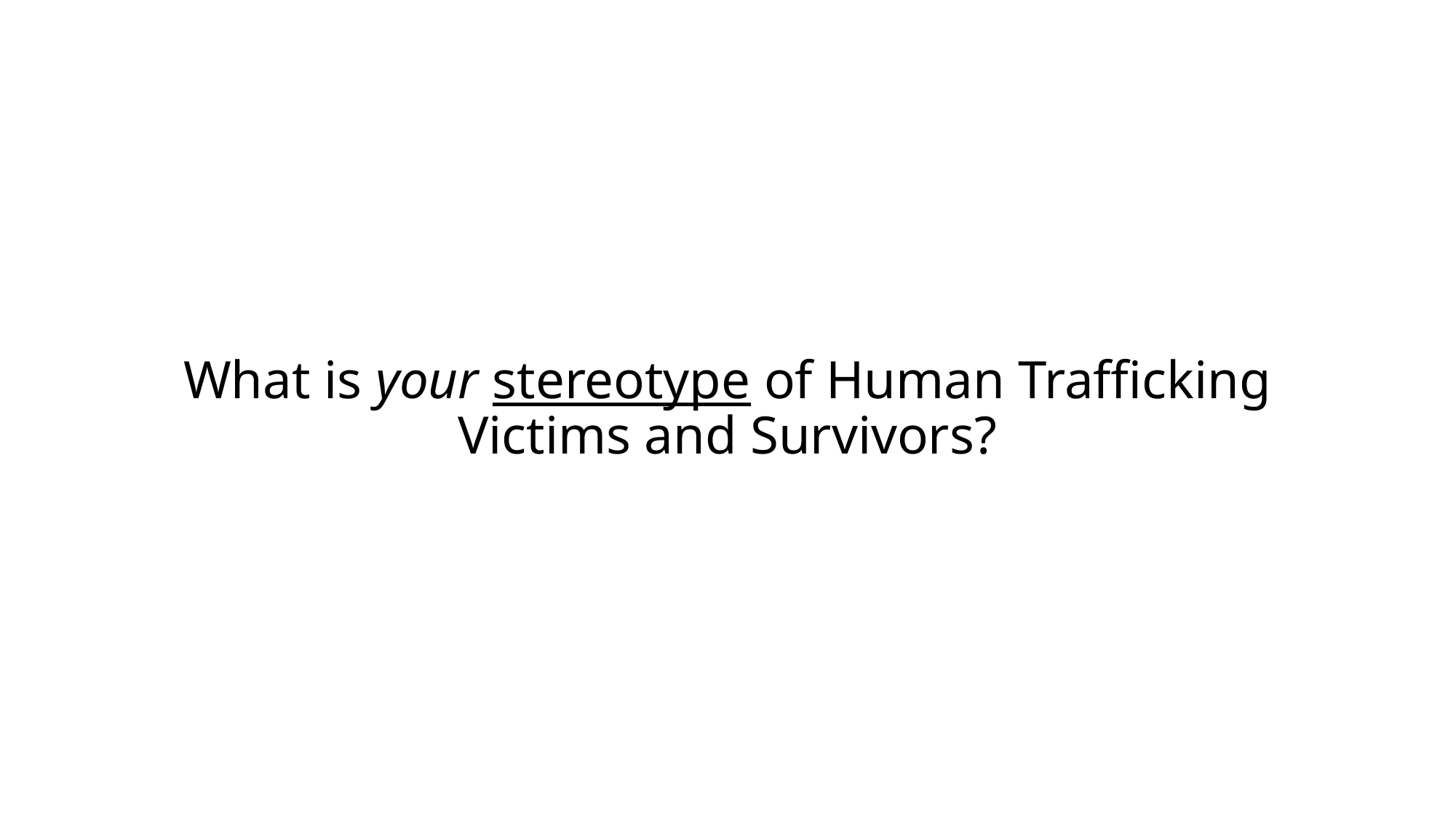

# What is your stereotype of Human Trafficking Victims and Survivors?

## Slide 6
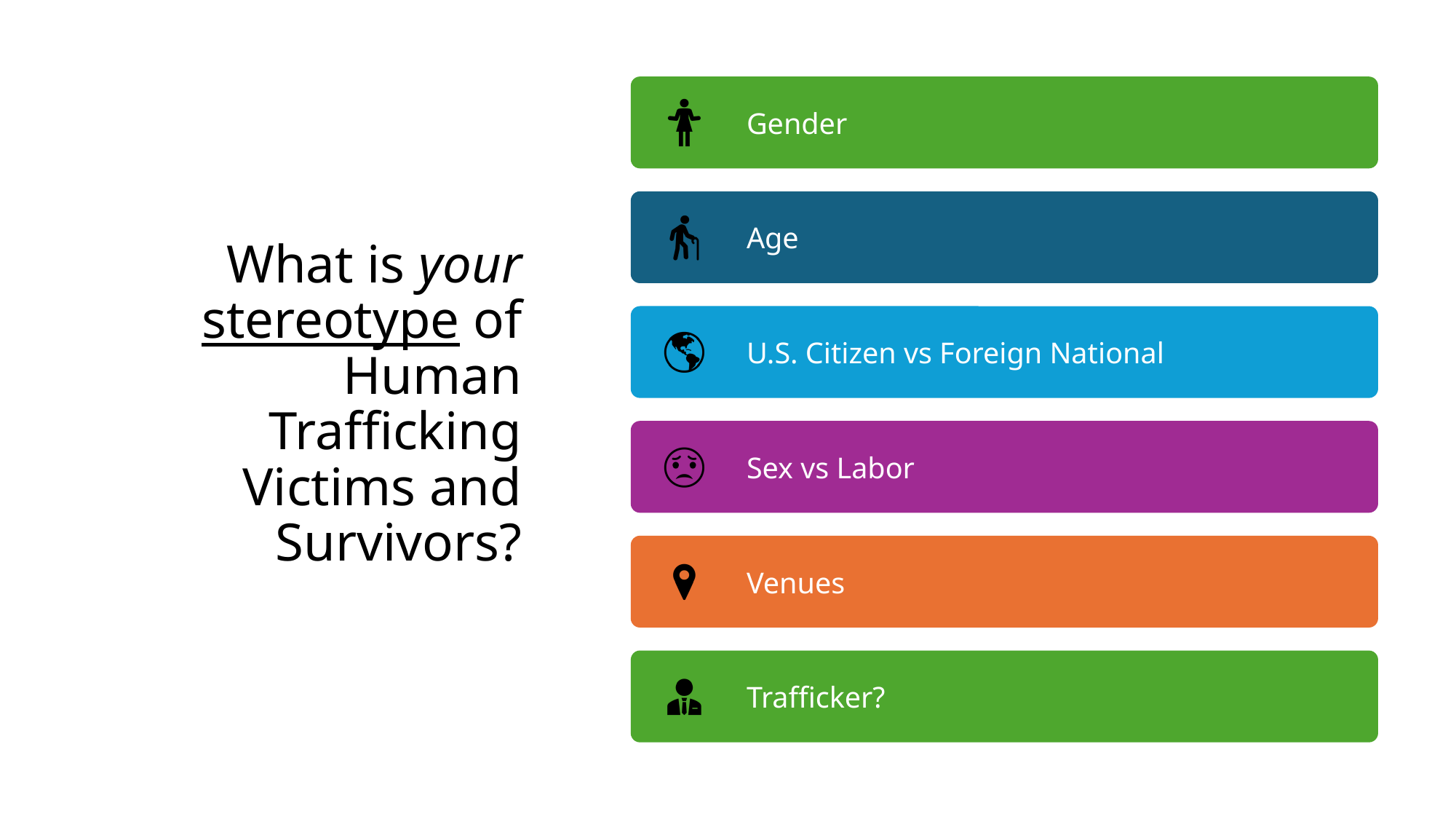

Gender
Age
U.S. Citizen vs Foreign National
Sex vs Labor
Venues
Trafficker?
# What is your stereotype of Human Trafficking Victims and Survivors?

## Slide 7
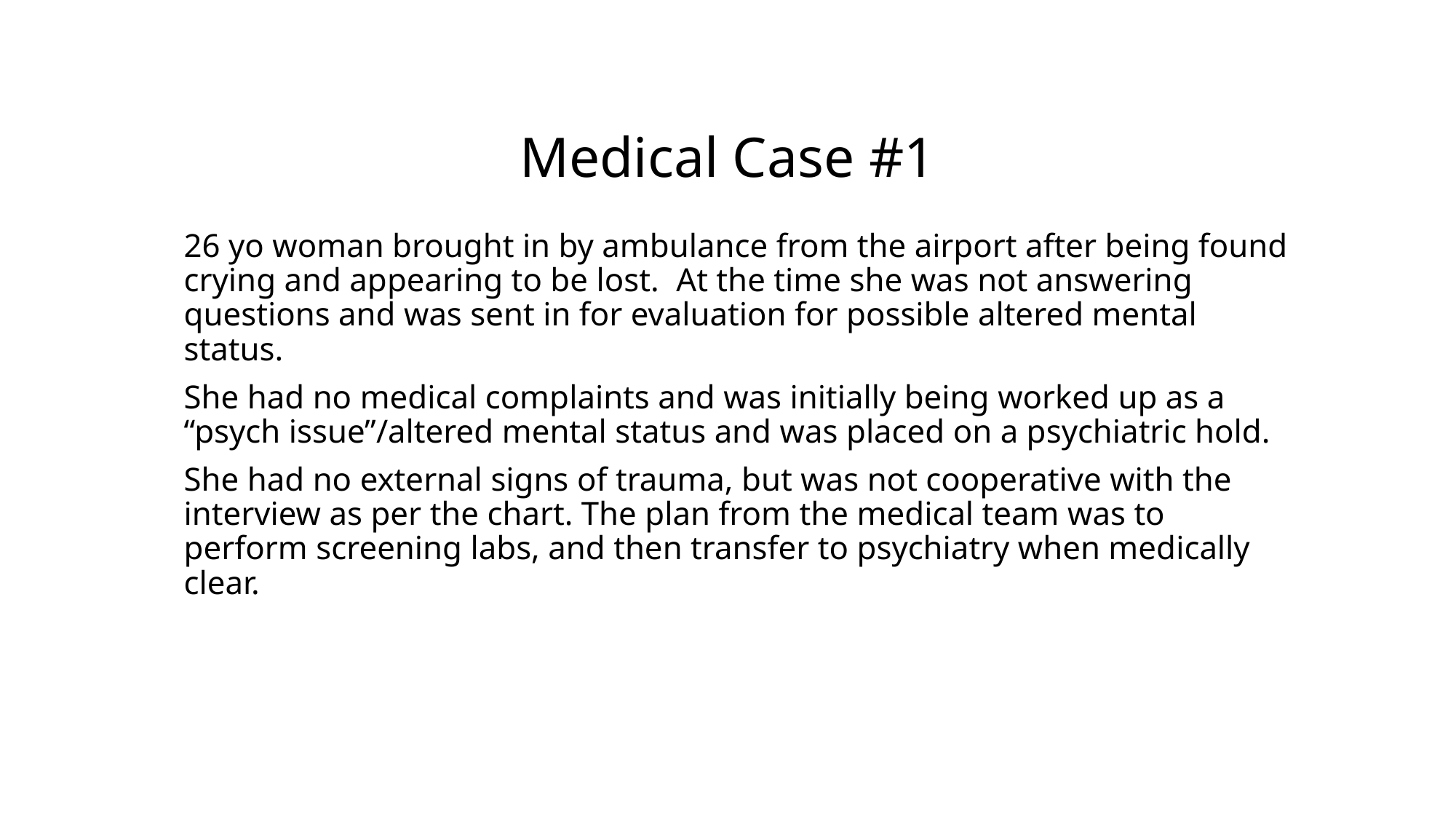

# Medical Case #1
26 yo woman brought in by ambulance from the airport after being found crying and appearing to be lost.  At the time she was not answering questions and was sent in for evaluation for possible altered mental status.
She had no medical complaints and was initially being worked up as a “psych issue”/altered mental status and was placed on a psychiatric hold.
She had no external signs of trauma, but was not cooperative with the interview as per the chart. The plan from the medical team was to perform screening labs, and then transfer to psychiatry when medically clear.

## Slide 8
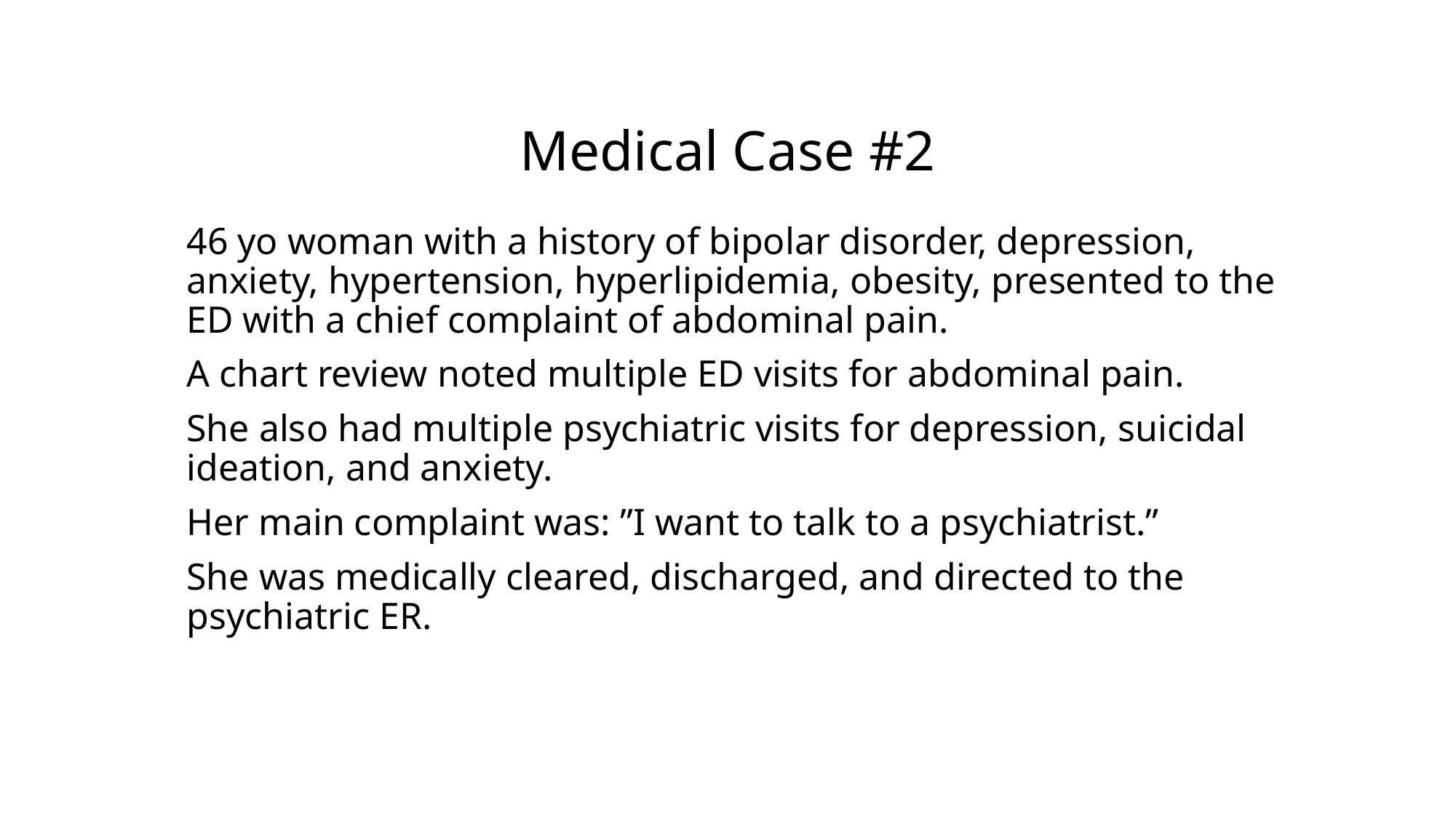

# Medical Case #2
46 yo woman with a history of bipolar disorder, depression, anxiety, hypertension, hyperlipidemia, obesity, presented to the ED with a chief complaint of abdominal pain.
A chart review noted multiple ED visits for abdominal pain.
She also had multiple psychiatric visits for depression, suicidal ideation, and anxiety.
Her main complaint was: ”I want to talk to a psychiatrist.”
She was medically cleared, discharged, and directed to the psychiatric ER.

## Slide 9
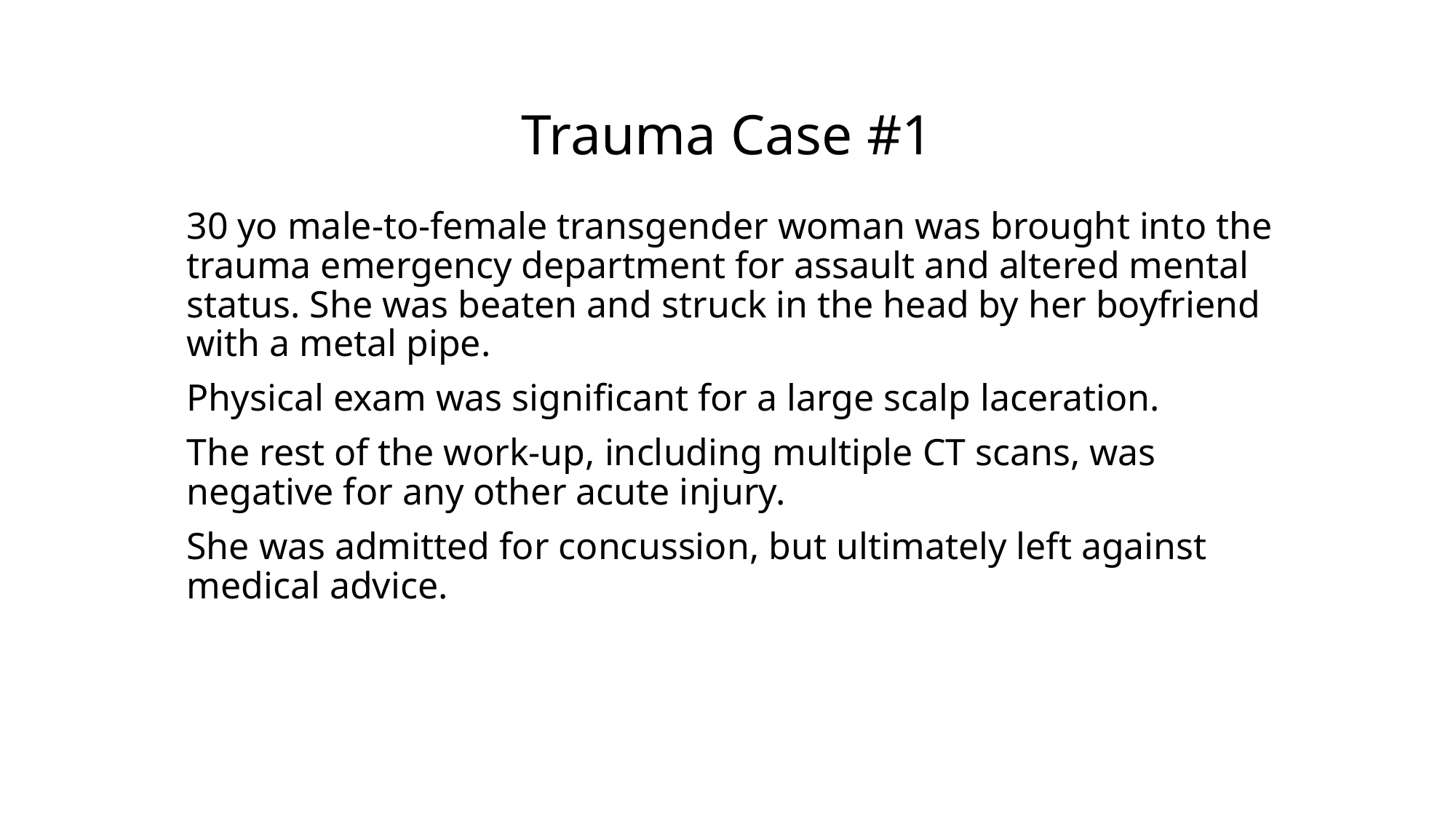

# Trauma Case #1
30 yo male-to-female transgender woman was brought into the trauma emergency department for assault and altered mental status. She was beaten and struck in the head by her boyfriend with a metal pipe.
Physical exam was significant for a large scalp laceration.
The rest of the work-up, including multiple CT scans, was negative for any other acute injury.
She was admitted for concussion, but ultimately left against medical advice.

## Slide 10
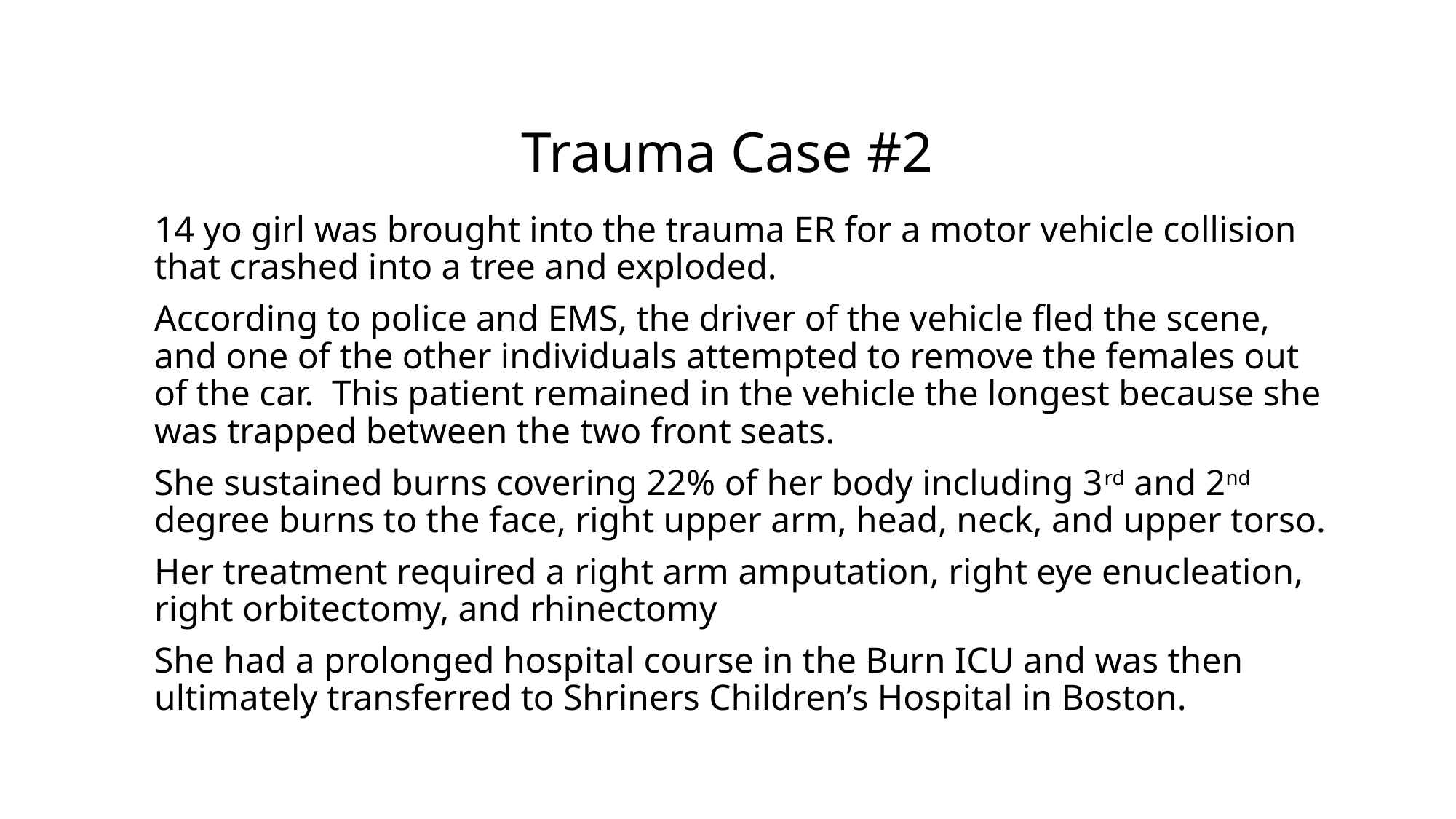

# Trauma Case #2
14 yo girl was brought into the trauma ER for a motor vehicle collision that crashed into a tree and exploded.
According to police and EMS, the driver of the vehicle fled the scene, and one of the other individuals attempted to remove the females out of the car. This patient remained in the vehicle the longest because she was trapped between the two front seats.
She sustained burns covering 22% of her body including 3rd and 2nd degree burns to the face, right upper arm, head, neck, and upper torso.
Her treatment required a right arm amputation, right eye enucleation, right orbitectomy, and rhinectomy
She had a prolonged hospital course in the Burn ICU and was then ultimately transferred to Shriners Children’s Hospital in Boston.

## Slide 11
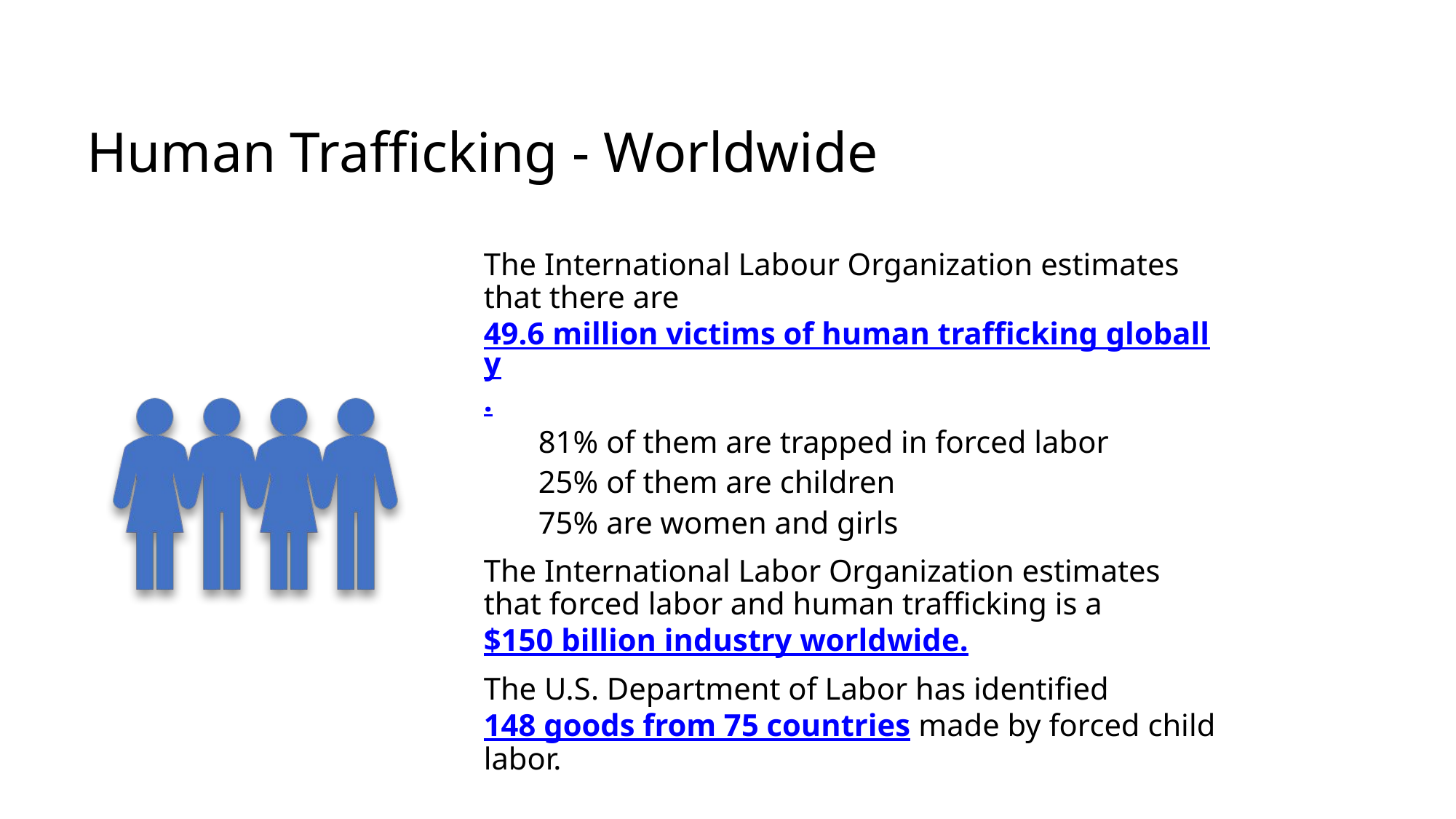

# Human Trafficking - Worldwide
The International Labour Organization estimates that there are 49.6 million victims of human trafficking globally.
81% of them are trapped in forced labor
25% of them are children
75% are women and girls
The International Labor Organization estimates that forced labor and human trafficking is a $150 billion industry worldwide.
The U.S. Department of Labor has identified 148 goods from 75 countries made by forced child labor.

## Slide 12
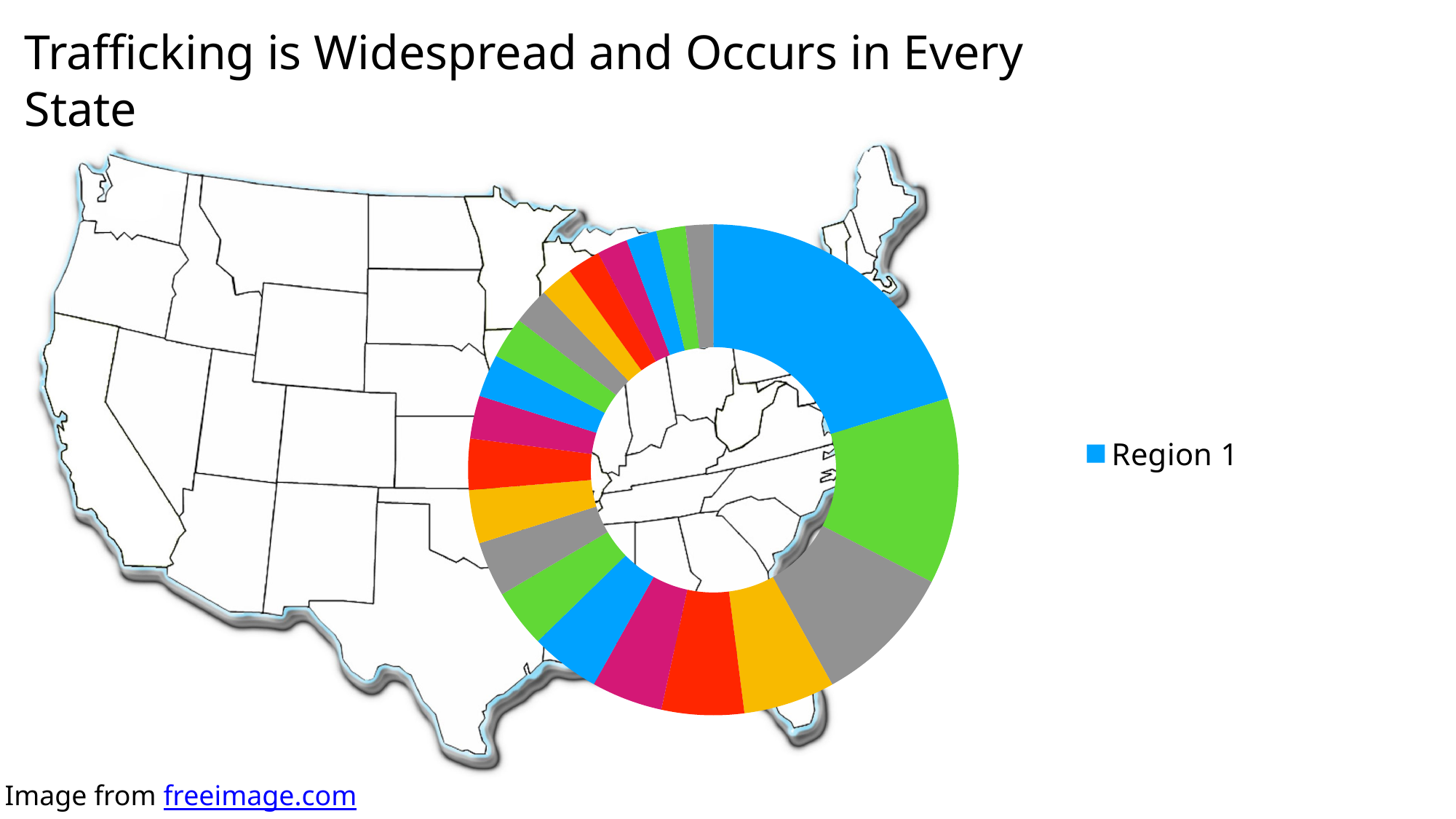

Trafficking is Widespread and Occurs in Every State
### Chart
| Category | Region 1 |
|---|---|
| California | 1656.0 |
| Texas | 1000.0 |
| Florida | 767.0 |
| New York | 492.0 |
| Ohio | 443.0 |
| Michigan | 383.0 |
| Georgia | 375.0 |
| Nevada | 313.0 |
| Illinois | 296.0 |
| N. Carolina | 287.0 |
| Pennsylvania | 275.0 |
| Arizona | 231.0 |
| Washington | 229.0 |
| New Jersey | 224.0 |
| Virginia | 198.0 |
| Missouri | 178.0 |
| Colorado | 178.0 |
| Tennessee | 165.0 |
| Maryland | 165.0 |
| S. Carolina | 156.0 |
| Louisiana | 149.0 |
Image from freeimage.com

## Slide 13
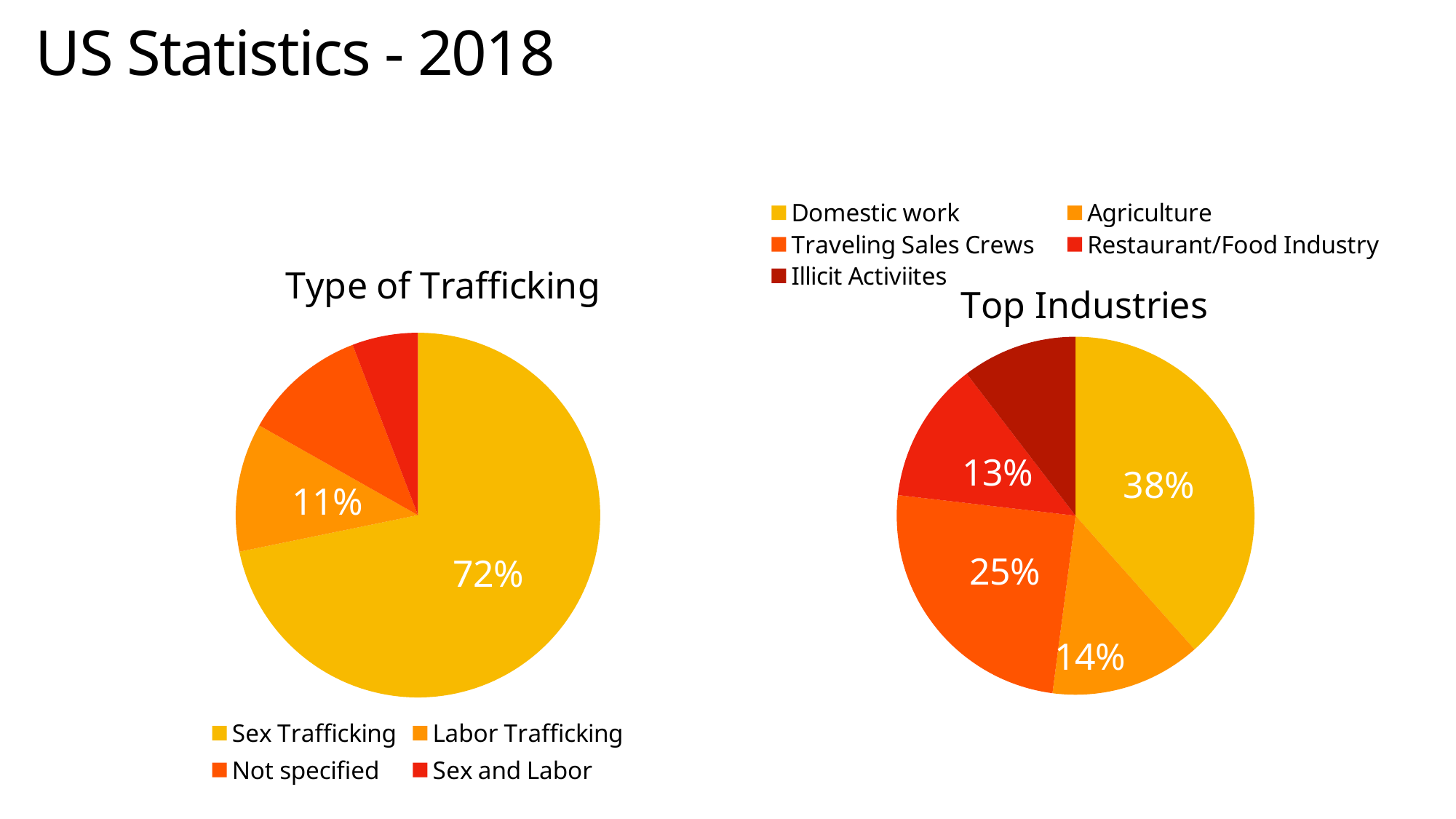

# US Statistics - 2018
### Chart: Top Industries
| Category | Region 1 |
|---|---|
| Domestic work | 214.0 |
| Agriculture | 76.0 |
| Traveling Sales Crews | 138.0 |
| Restaurant/Food Industry | 71.0 |
| Illicit Activiites | 58.0 |
### Chart: Type of Trafficking
| Category | Region 1 |
|---|---|
| Sex Trafficking | 7859.0 |
| Labor Trafficking | 1249.0 |
| Not specified | 1202.0 |
| Sex and Labor | 639.0 |

## Slide 14
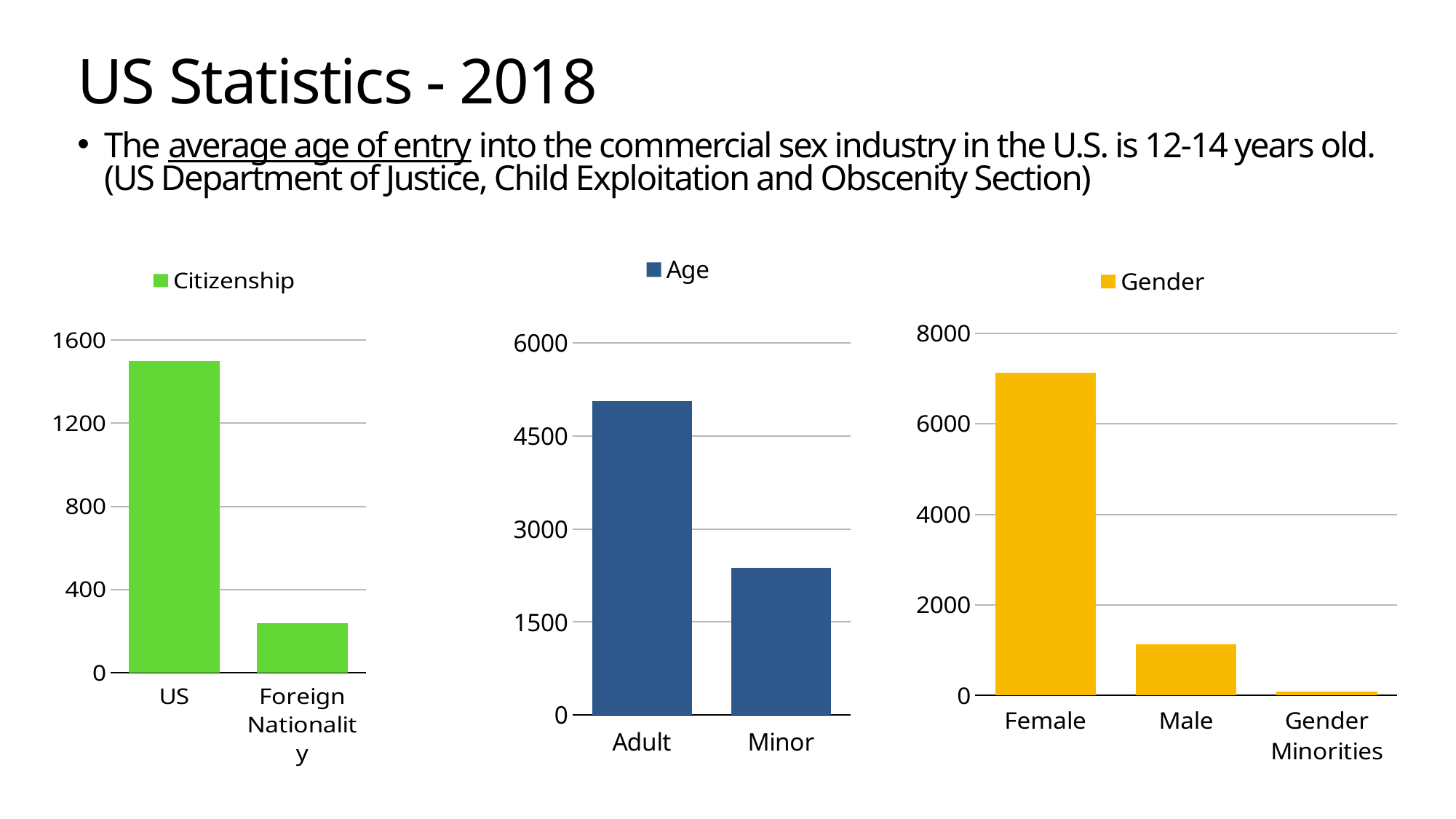

# US Statistics - 2018
The average age of entry into the commercial sex industry in the U.S. is 12-14 years old. (US Department of Justice, Child Exploitation and Obscenity Section)
### Chart
| Category | Age |
|---|---|
| Adult | 5070.0 |
| Minor | 2378.0 |
### Chart
| Category | Citizenship |
|---|---|
| US | 1499.0 |
| Foreign Nationality | 237.0 |
### Chart
| Category | Gender |
|---|---|
| Female | 7126.0 |
| Male | 1137.0 |
| Gender Minorities | 78.0 |

## Slide 15
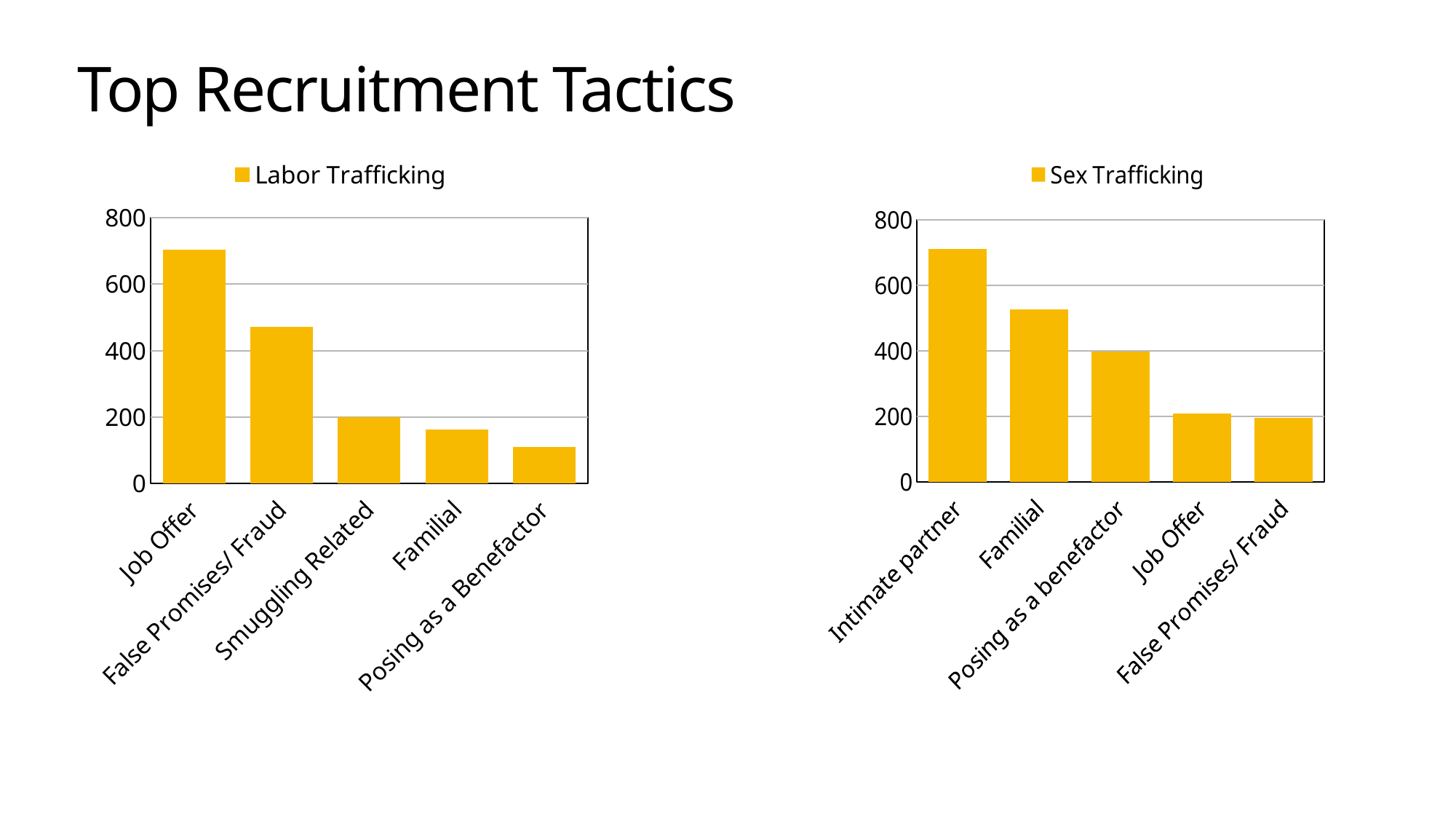

# Top Recruitment Tactics
### Chart
| Category | Labor Trafficking |
|---|---|
| Job Offer | 704.0 |
| False Promises/ Fraud | 471.0 |
| Smuggling Related | 200.0 |
| Familial | 162.0 |
| Posing as a Benefactor | 110.0 |
### Chart
| Category | Sex Trafficking |
|---|---|
| Intimate partner | 711.0 |
| Familial | 525.0 |
| Posing as a benefactor | 397.0 |
| Job Offer | 209.0 |
| False Promises/ Fraud | 195.0 |

## Slide 16
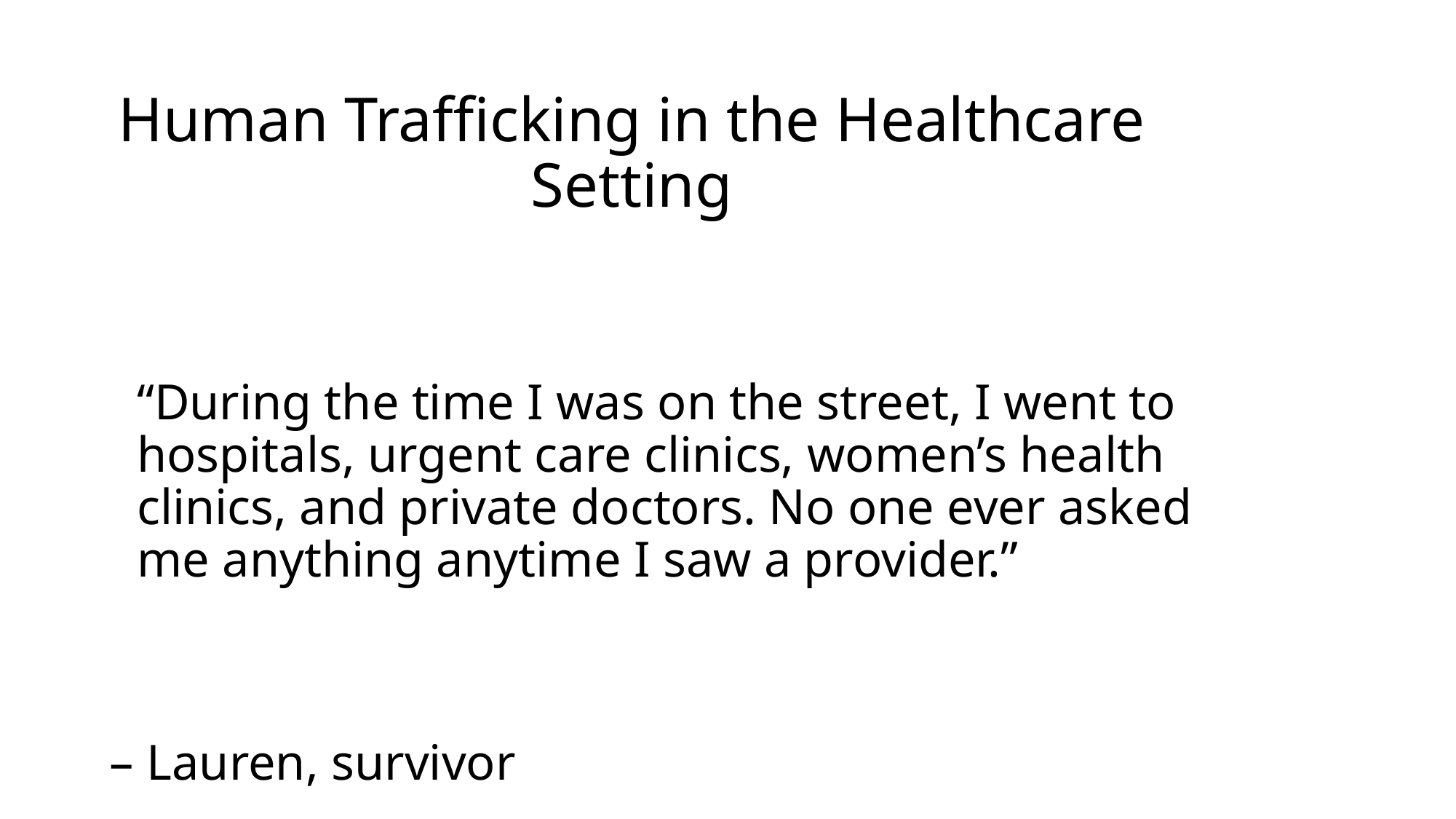

# Human Trafficking in the Healthcare Setting
“During the time I was on the street, I went to hospitals, urgent care clinics, women’s health clinics, and private doctors. No one ever asked me anything anytime I saw a provider.”
– Lauren, survivor

## Slide 17
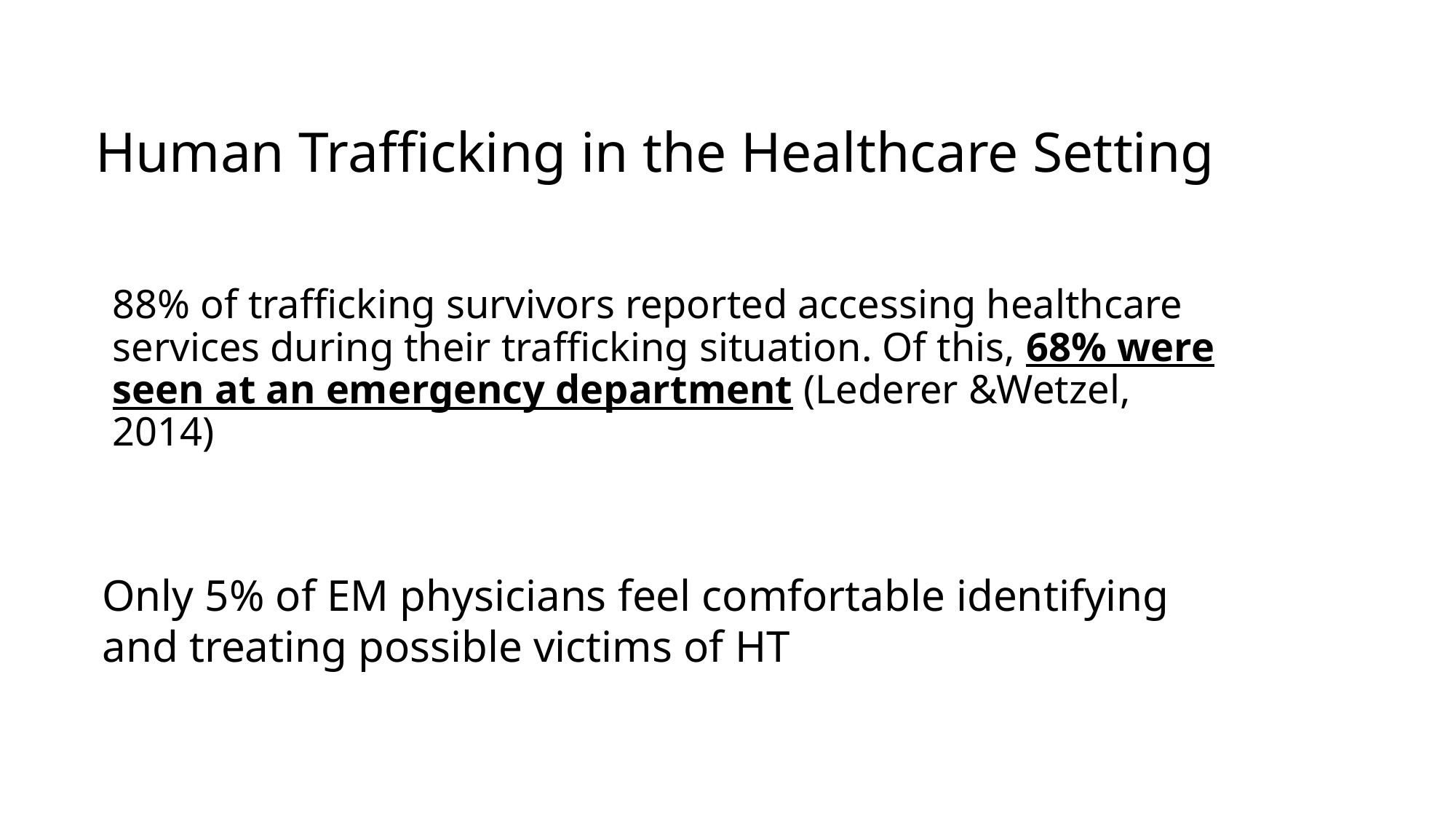

# Human Trafficking in the Healthcare Setting
88% of trafficking survivors reported accessing healthcare services during their trafficking situation. Of this, 68% were seen at an emergency department (Lederer &Wetzel, 2014)
Only 5% of EM physicians feel comfortable identifying and treating possible victims of HT

## Slide 18
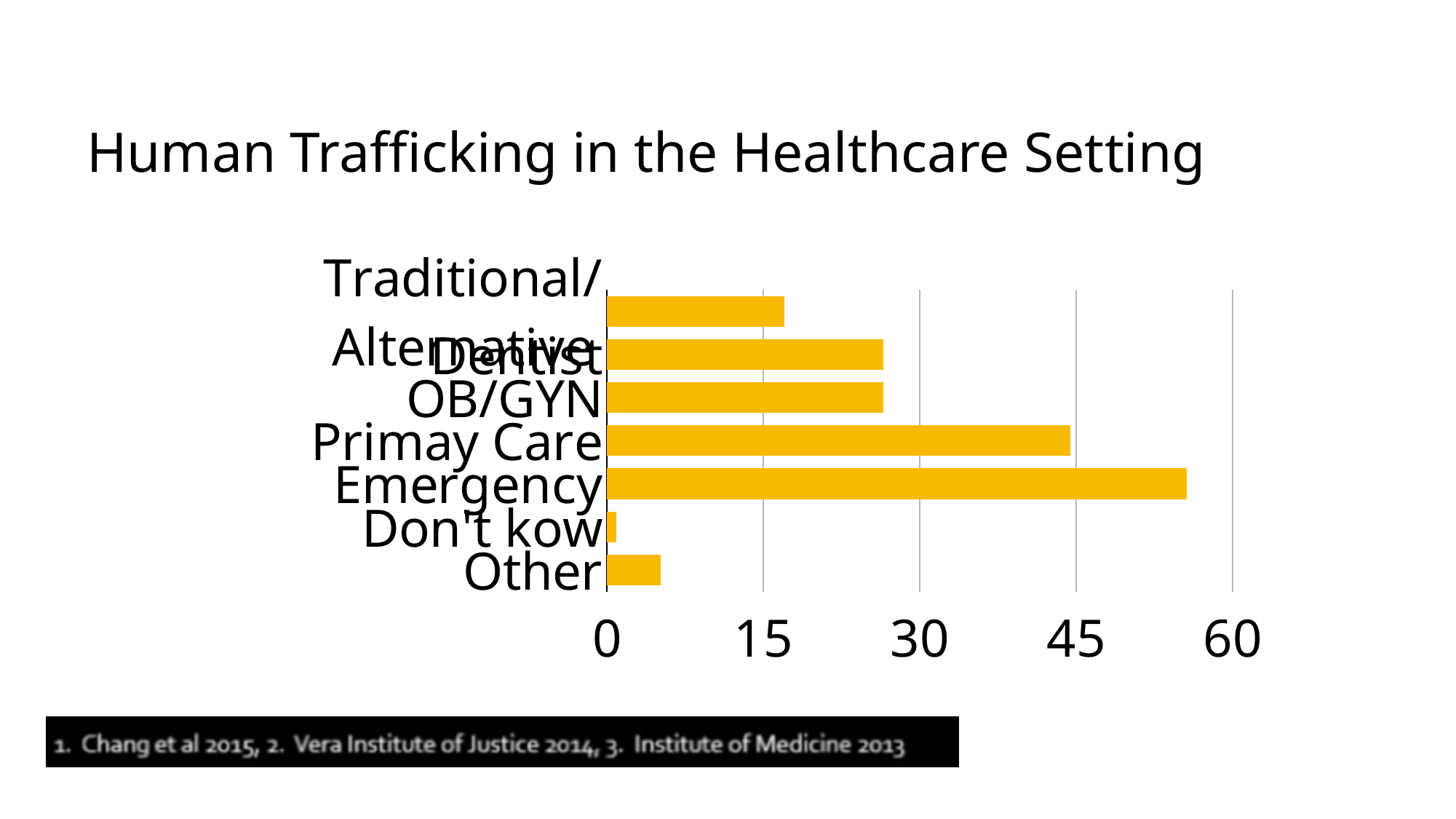

# Human Trafficking in the Healthcare Setting
### Chart
| Category | |
|---|---|
| Traditional/Alternative | 17.0 |
| Dentist | 26.5 |
| OB/GYN | 26.5 |
| Primay Care | 44.4 |
| Emergency | 55.6 |
| Don't kow | 0.9 |
| Other | 5.1 |

## Slide 19
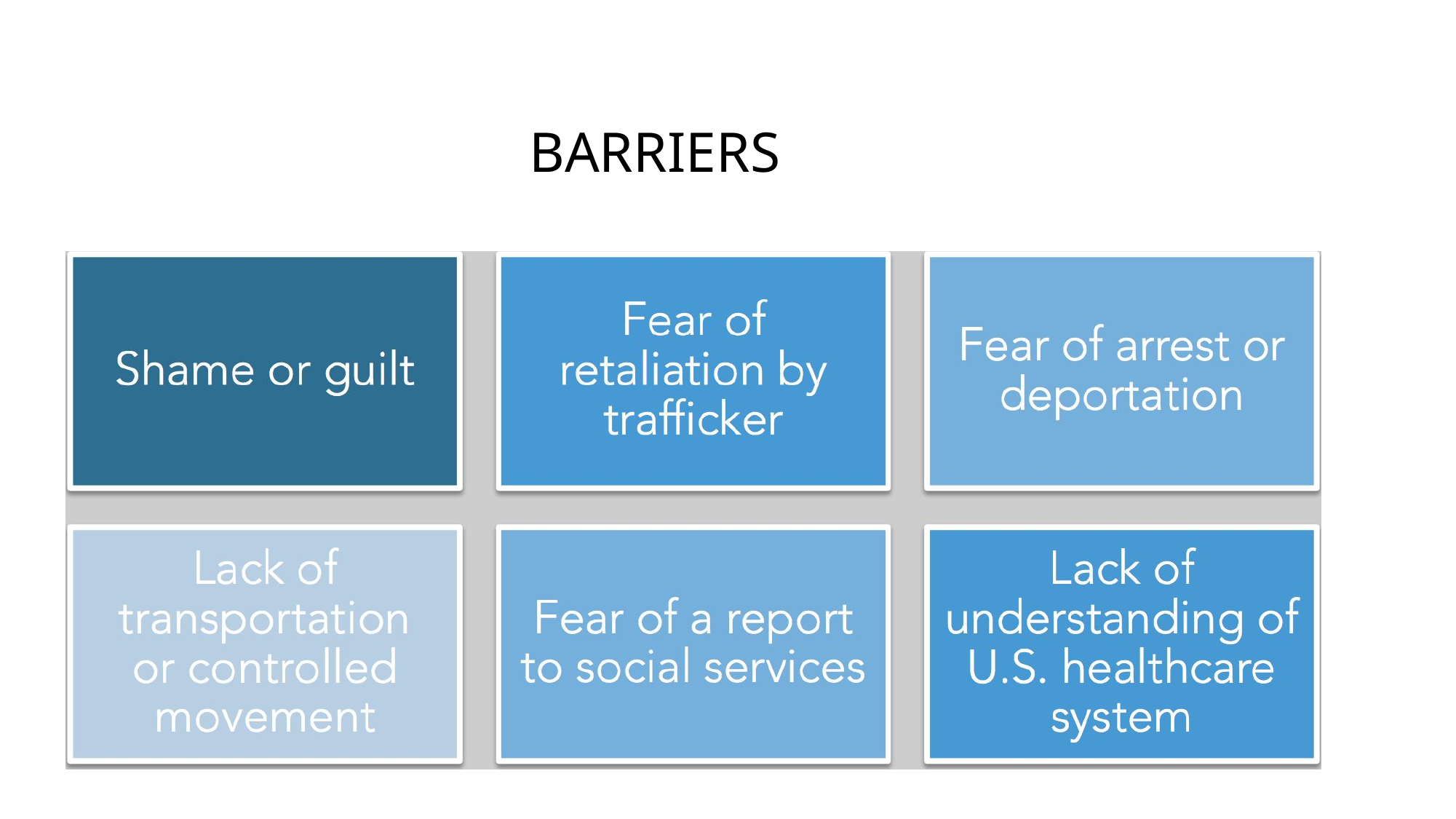

# BARRIERS

## Slide 20
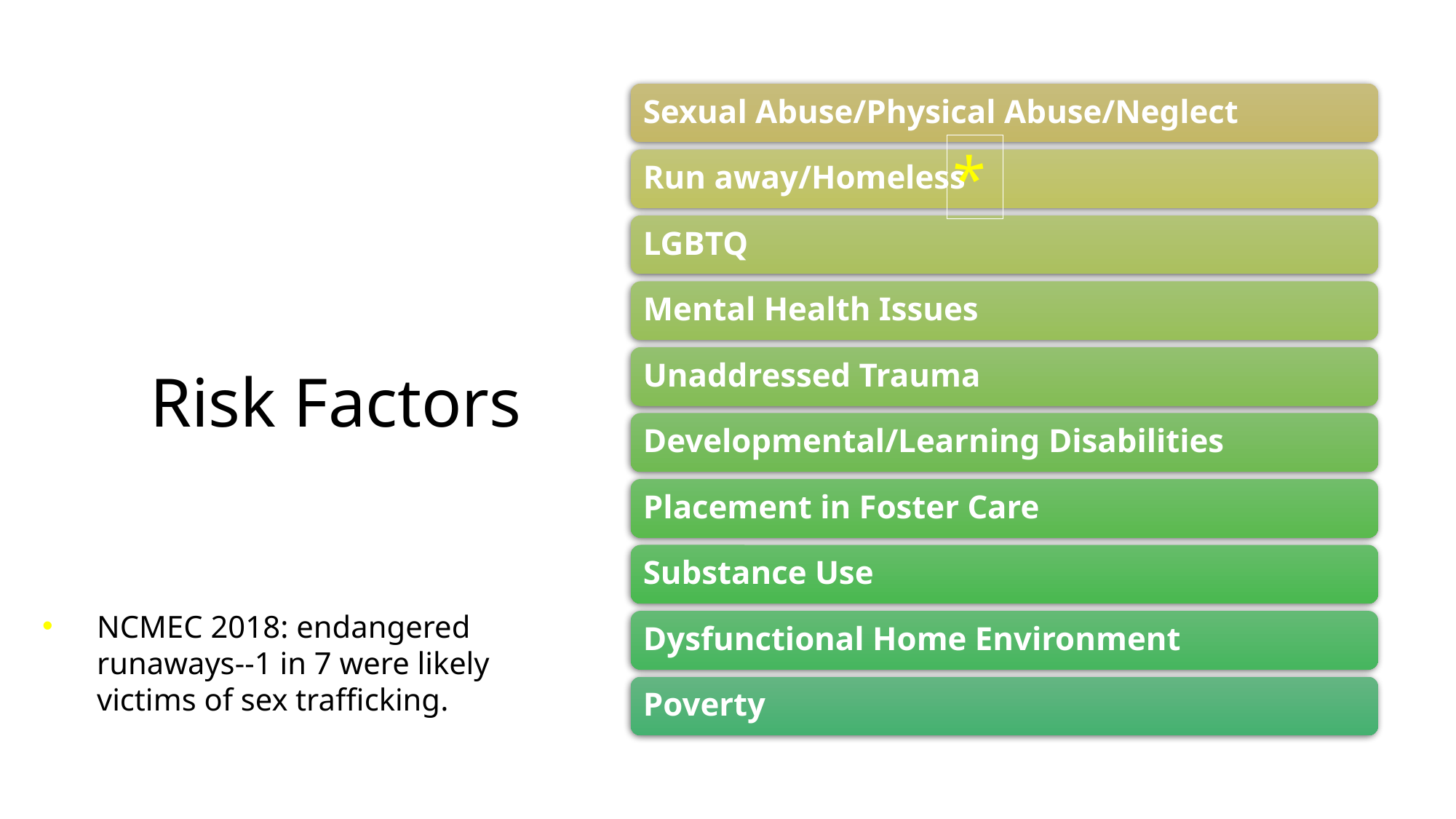

Sexual Abuse/Physical Abuse/Neglect
Run away/Homeless
LGBTQ
Mental Health Issues
Unaddressed Trauma
Developmental/Learning Disabilities
Placement in Foster Care
Substance Use
Dysfunctional Home Environment
Poverty
*
# Risk Factors
NCMEC 2018: endangered runaways--1 in 7 were likely victims of sex trafficking.

## Slide 21
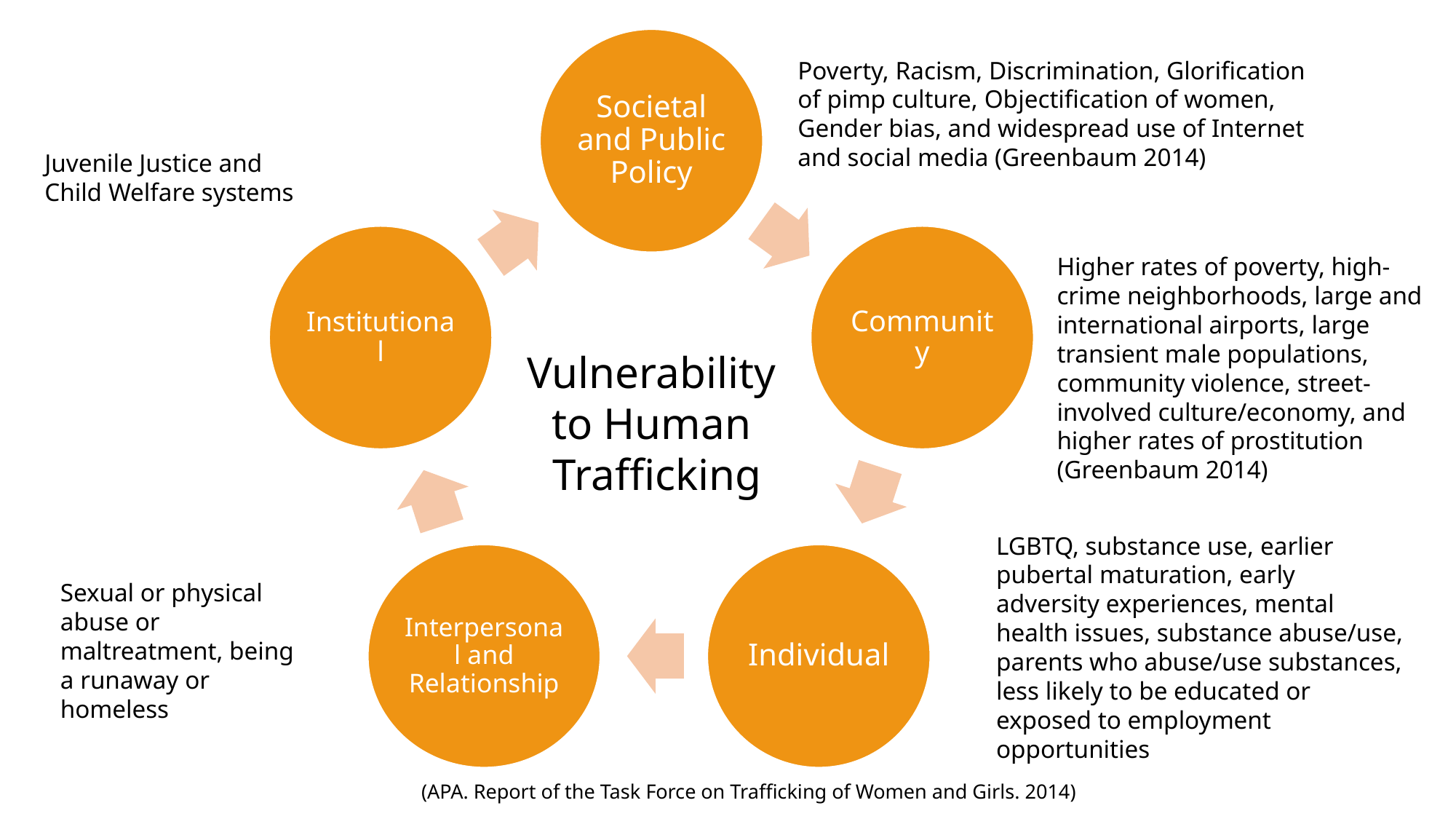

Societal and Public Policy
Community
Institutional
Interpersonal and Relationship
Individual
Poverty, Racism, Discrimination, Glorification of pimp culture, Objectification of women, Gender bias, and widespread use of Internet and social media (Greenbaum 2014)
Juvenile Justice and Child Welfare systems
Higher rates of poverty, high-crime neighborhoods, large and international airports, large transient male populations, community violence, street-involved culture/economy, and higher rates of prostitution (Greenbaum 2014)
Vulnerability to Human
 Trafficking
LGBTQ, substance use, earlier pubertal maturation, early adversity experiences, mental health issues, substance abuse/use, parents who abuse/use substances, less likely to be educated or exposed to employment opportunities
Sexual or physical abuse or maltreatment, being a runaway or homeless
		(APA. Report of the Task Force on Trafficking of Women and Girls. 2014)

## Slide 22
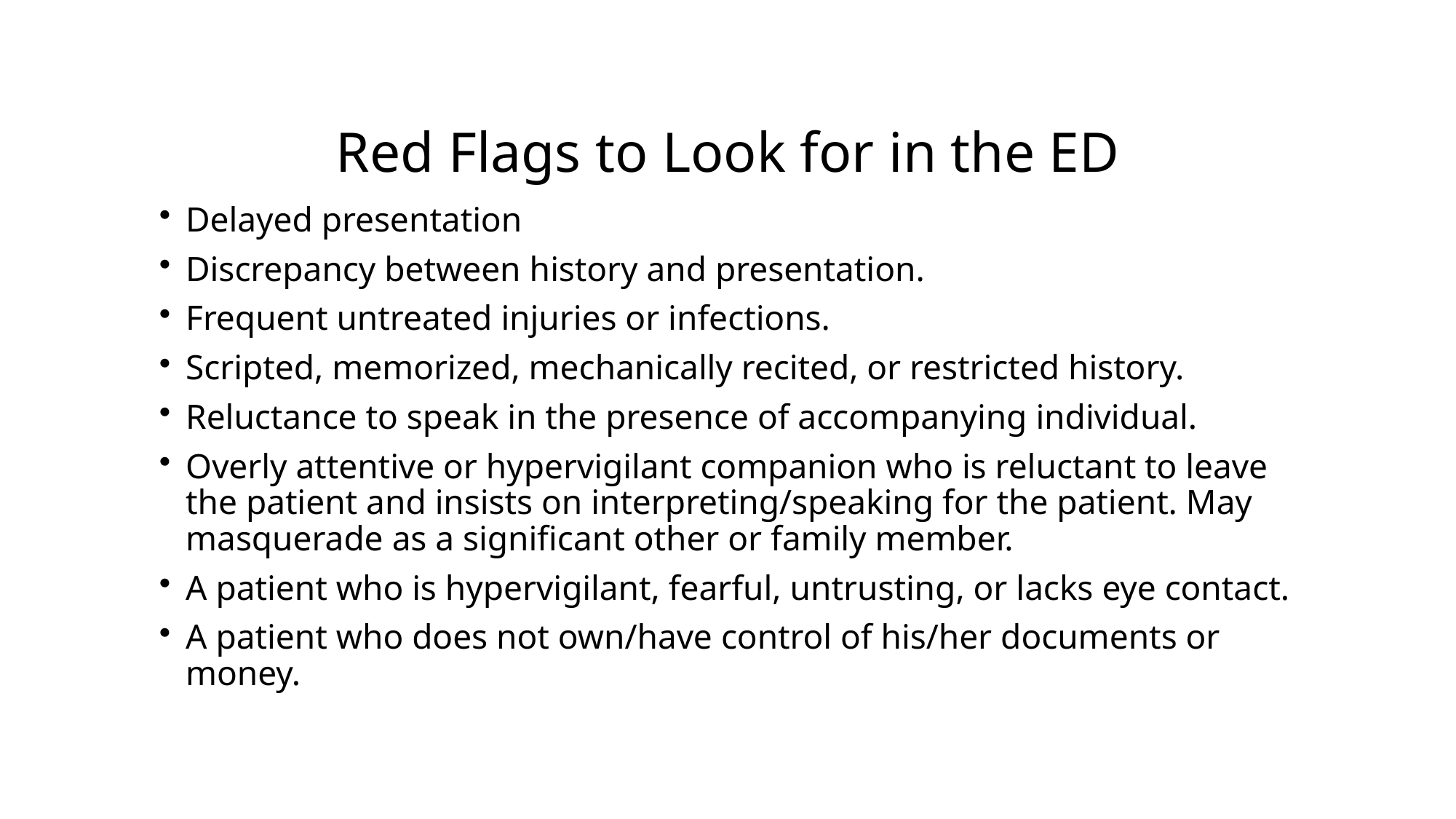

# Red Flags to Look for in the ED
Delayed presentation
Discrepancy between history and presentation.
Frequent untreated injuries or infections.
Scripted, memorized, mechanically recited, or restricted history.
Reluctance to speak in the presence of accompanying individual.
Overly attentive or hypervigilant companion who is reluctant to leave the patient and insists on interpreting/speaking for the patient. May masquerade as a significant other or family member.
A patient who is hypervigilant, fearful, untrusting, or lacks eye contact.
A patient who does not own/have control of his/her documents or money.

## Slide 23
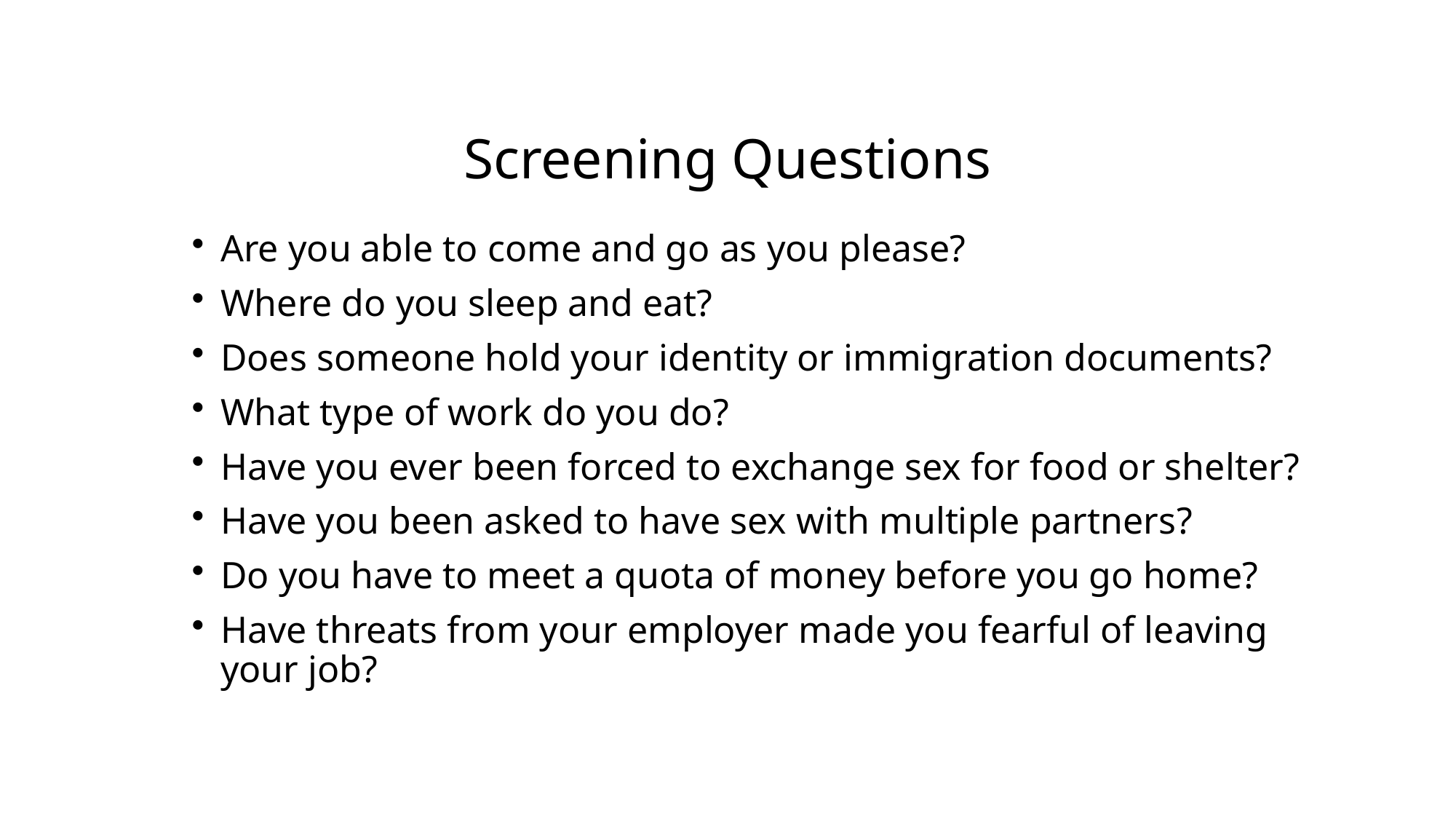

# Screening Questions
Are you able to come and go as you please?
Where do you sleep and eat?
Does someone hold your identity or immigration documents?
What type of work do you do?
Have you ever been forced to exchange sex for food or shelter?
Have you been asked to have sex with multiple partners?
Do you have to meet a quota of money before you go home?
Have threats from your employer made you fearful of leaving your job?

## Slide 24
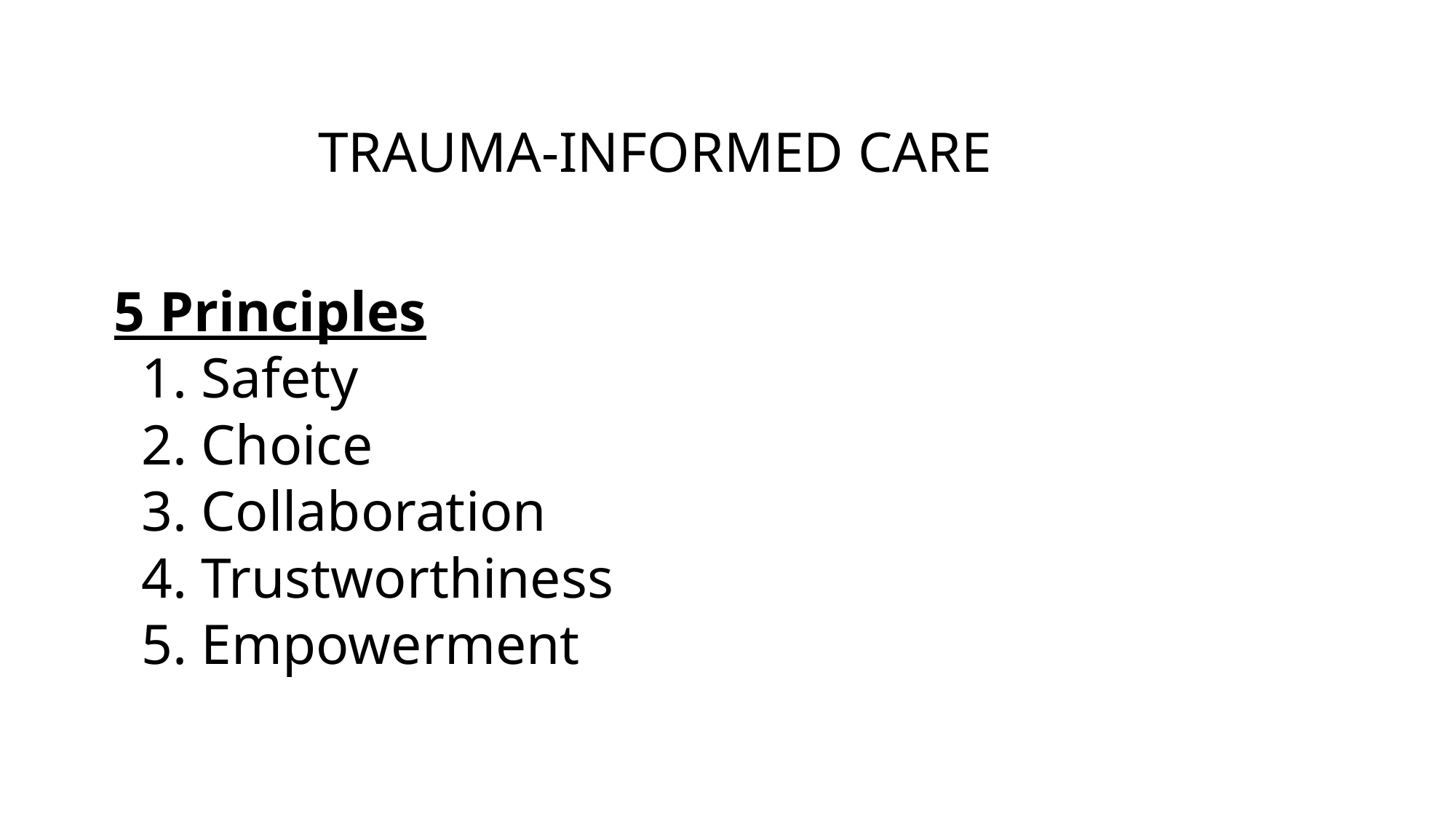

# TRAUMA-INFORMED CARE
5 Principles
1. Safety
2. Choice
3. Collaboration
4. Trustworthiness
5. Empowerment

## Slide 25
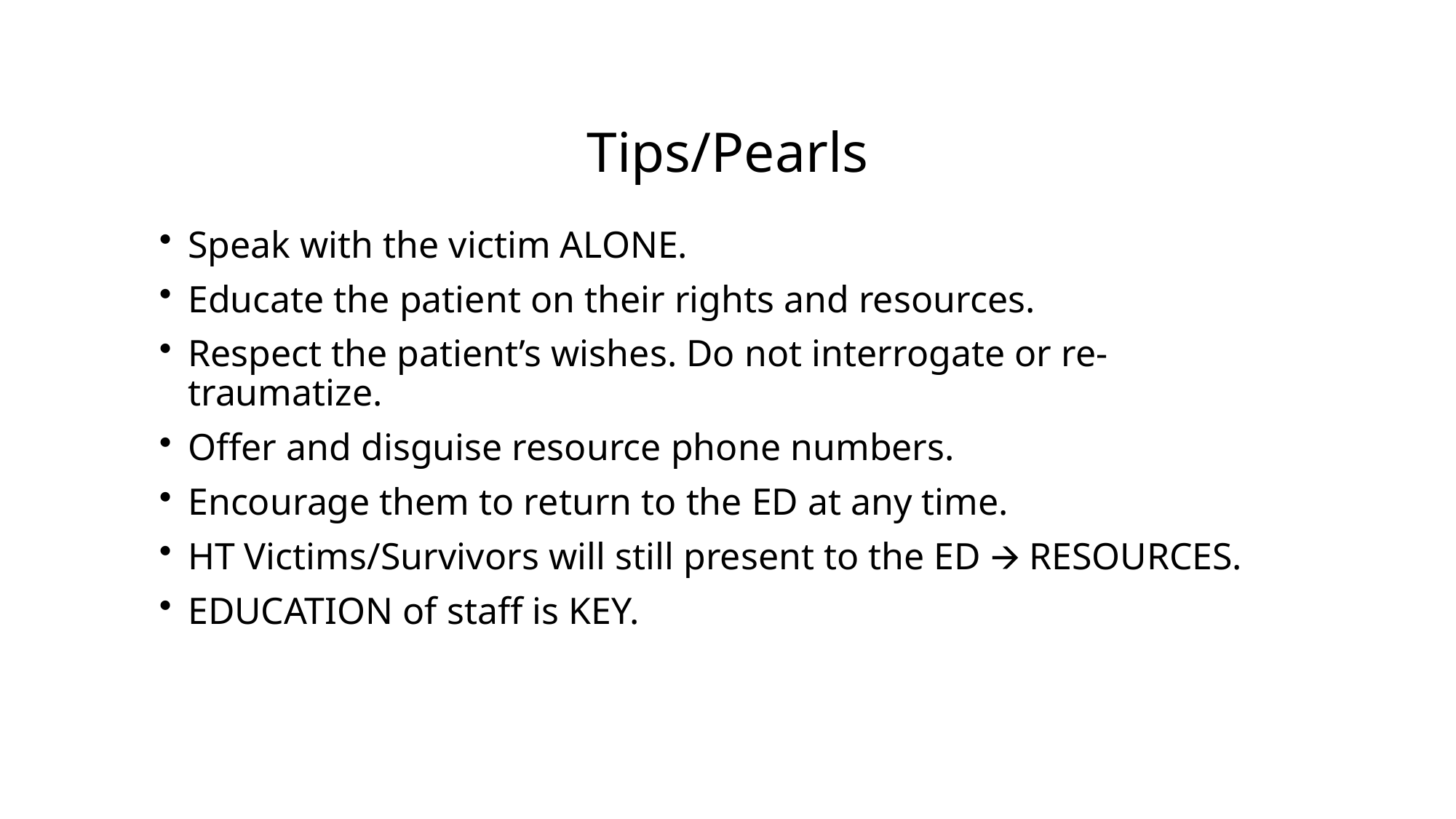

# Tips/Pearls
Speak with the victim ALONE.
Educate the patient on their rights and resources.
Respect the patient’s wishes. Do not interrogate or re-traumatize.
Offer and disguise resource phone numbers.
Encourage them to return to the ED at any time.
HT Victims/Survivors will still present to the ED 🡪 RESOURCES.
EDUCATION of staff is KEY.

## Slide 26
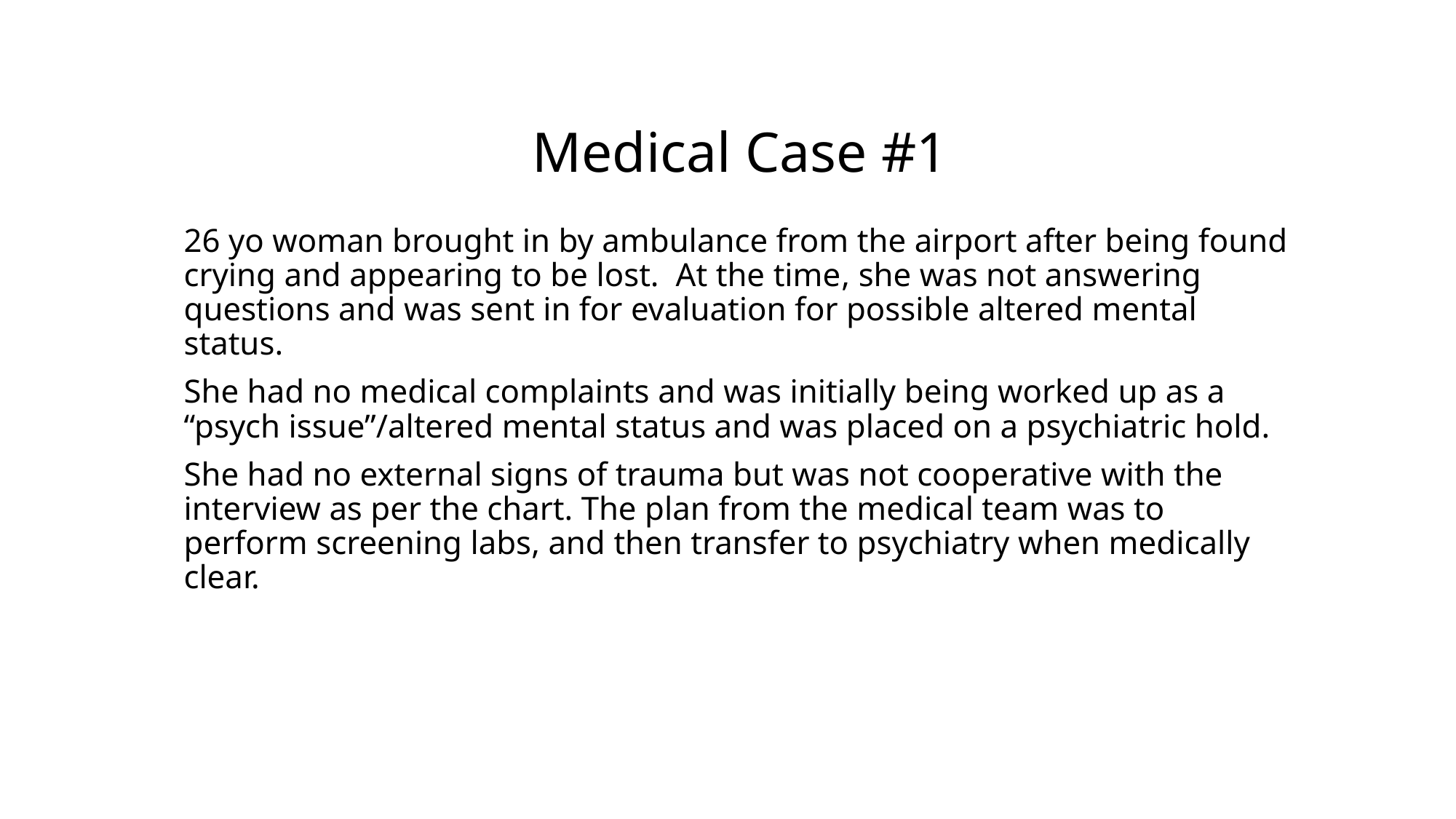

# Medical Case #1
26 yo woman brought in by ambulance from the airport after being found crying and appearing to be lost. At the time, she was not answering questions and was sent in for evaluation for possible altered mental status.
She had no medical complaints and was initially being worked up as a “psych issue”/altered mental status and was placed on a psychiatric hold.
She had no external signs of trauma but was not cooperative with the interview as per the chart. The plan from the medical team was to perform screening labs, and then transfer to psychiatry when medically clear.

## Slide 27
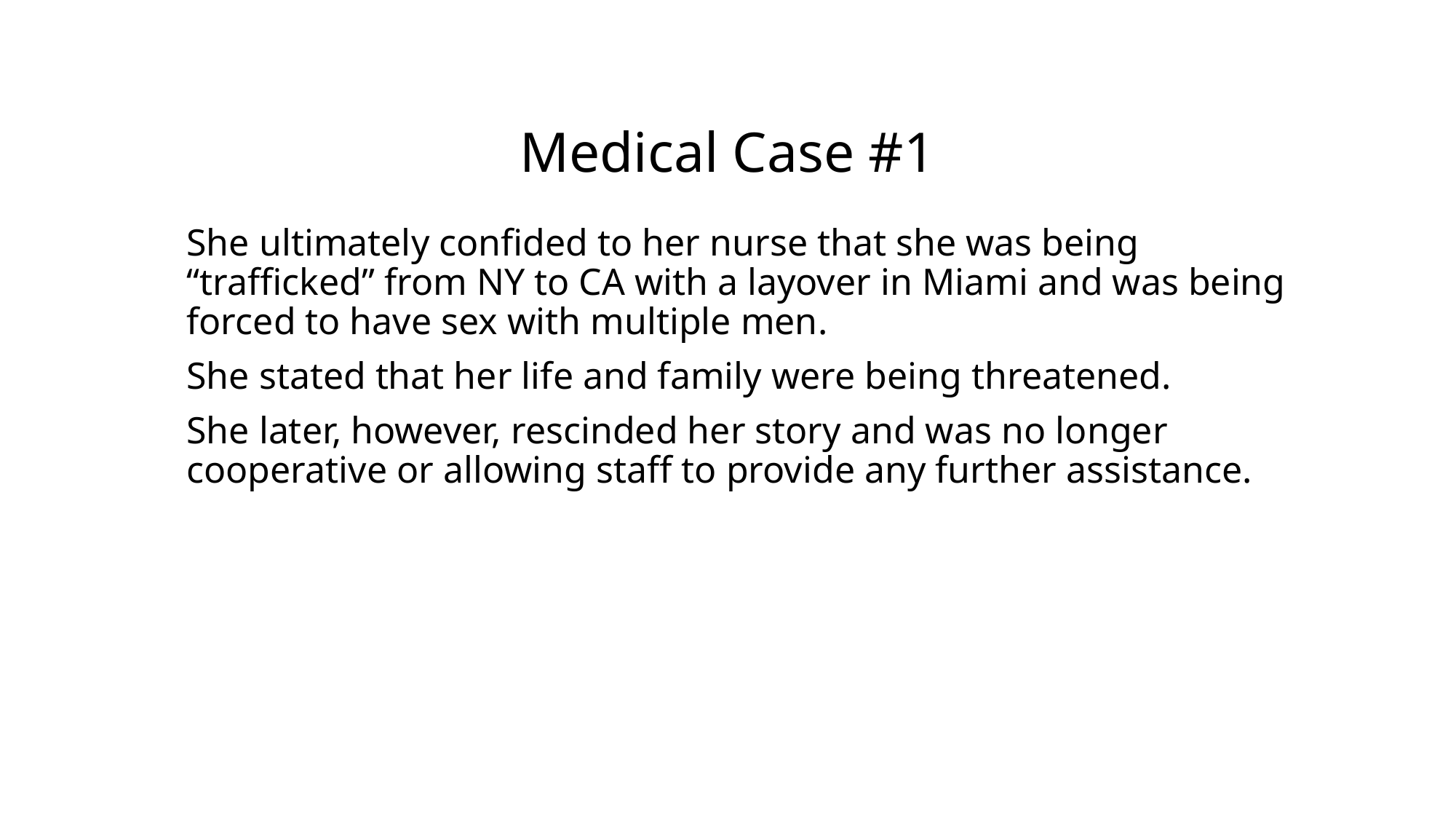

# Medical Case #1
She ultimately confided to her nurse that she was being “trafficked” from NY to CA with a layover in Miami and was being forced to have sex with multiple men.
She stated that her life and family were being threatened.
She later, however, rescinded her story and was no longer cooperative or allowing staff to provide any further assistance.

## Slide 28
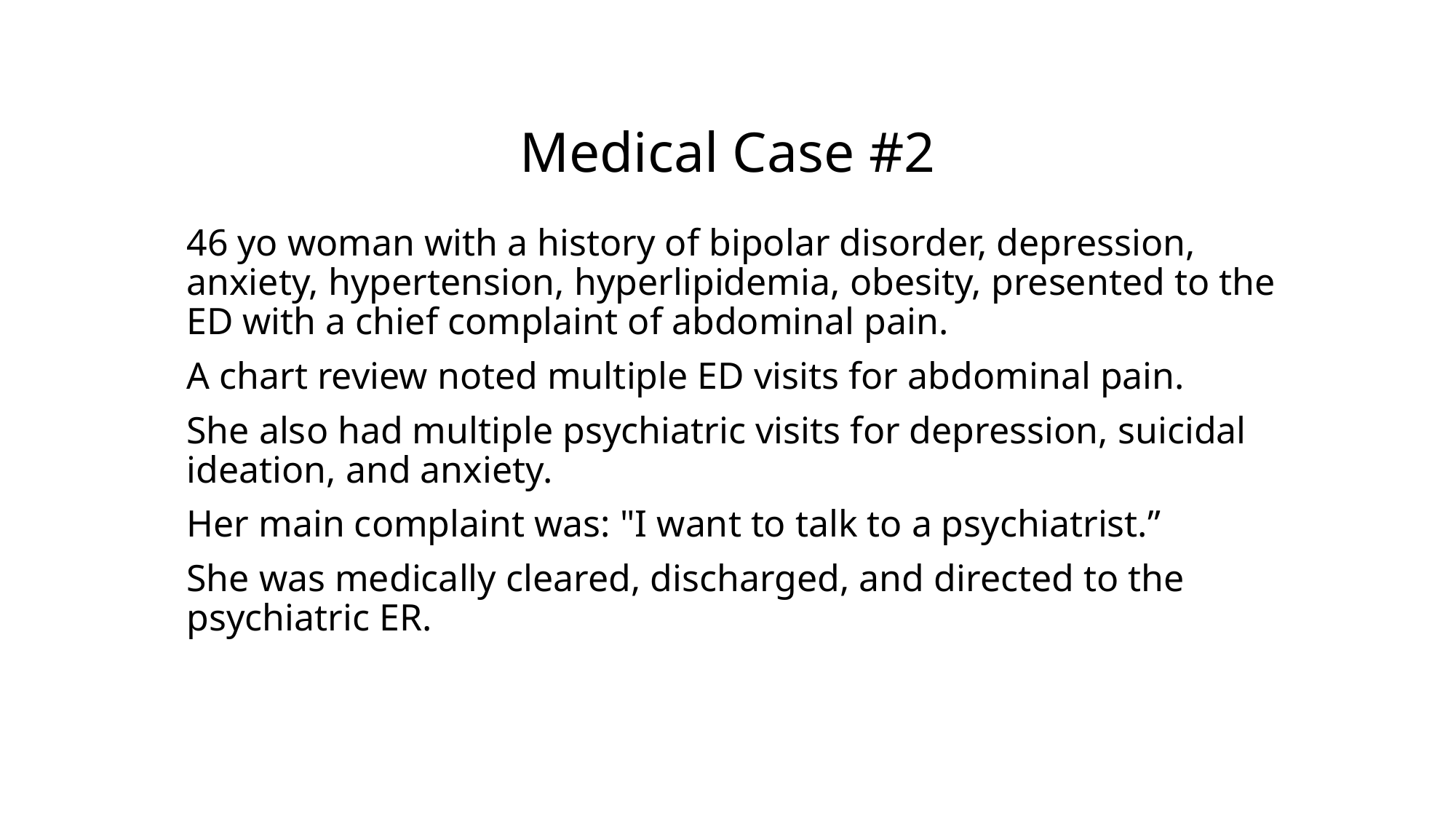

# Medical Case #2
46 yo woman with a history of bipolar disorder, depression, anxiety, hypertension, hyperlipidemia, obesity, presented to the ED with a chief complaint of abdominal pain.
A chart review noted multiple ED visits for abdominal pain.
She also had multiple psychiatric visits for depression, suicidal ideation, and anxiety.
Her main complaint was: "I want to talk to a psychiatrist.”
She was medically cleared, discharged, and directed to the psychiatric ER.

## Slide 29
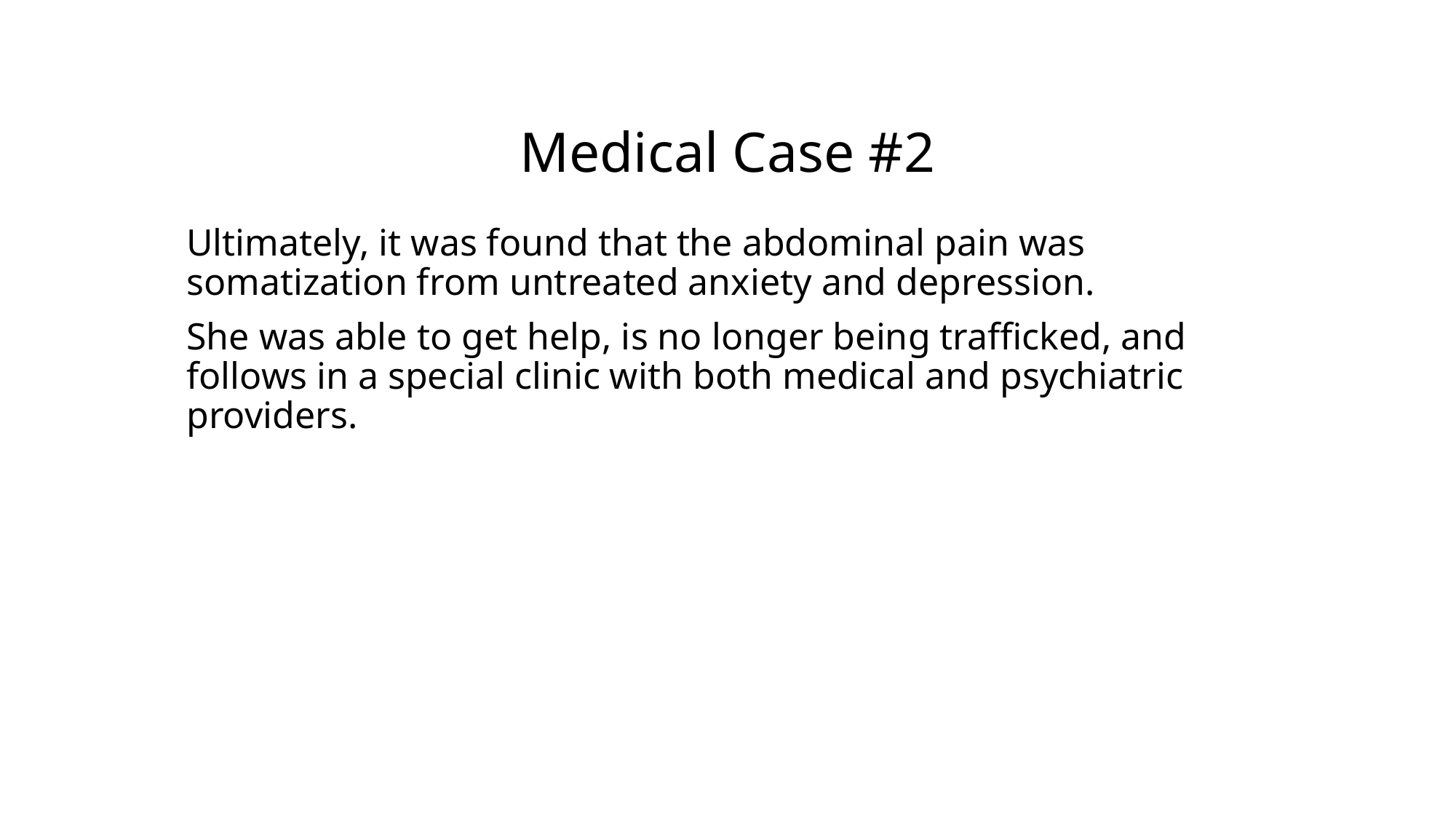

# Medical Case #2
Ultimately, it was found that the abdominal pain was somatization from untreated anxiety and depression.
She was able to get help, is no longer being trafficked, and follows in a special clinic with both medical and psychiatric providers.

## Slide 30
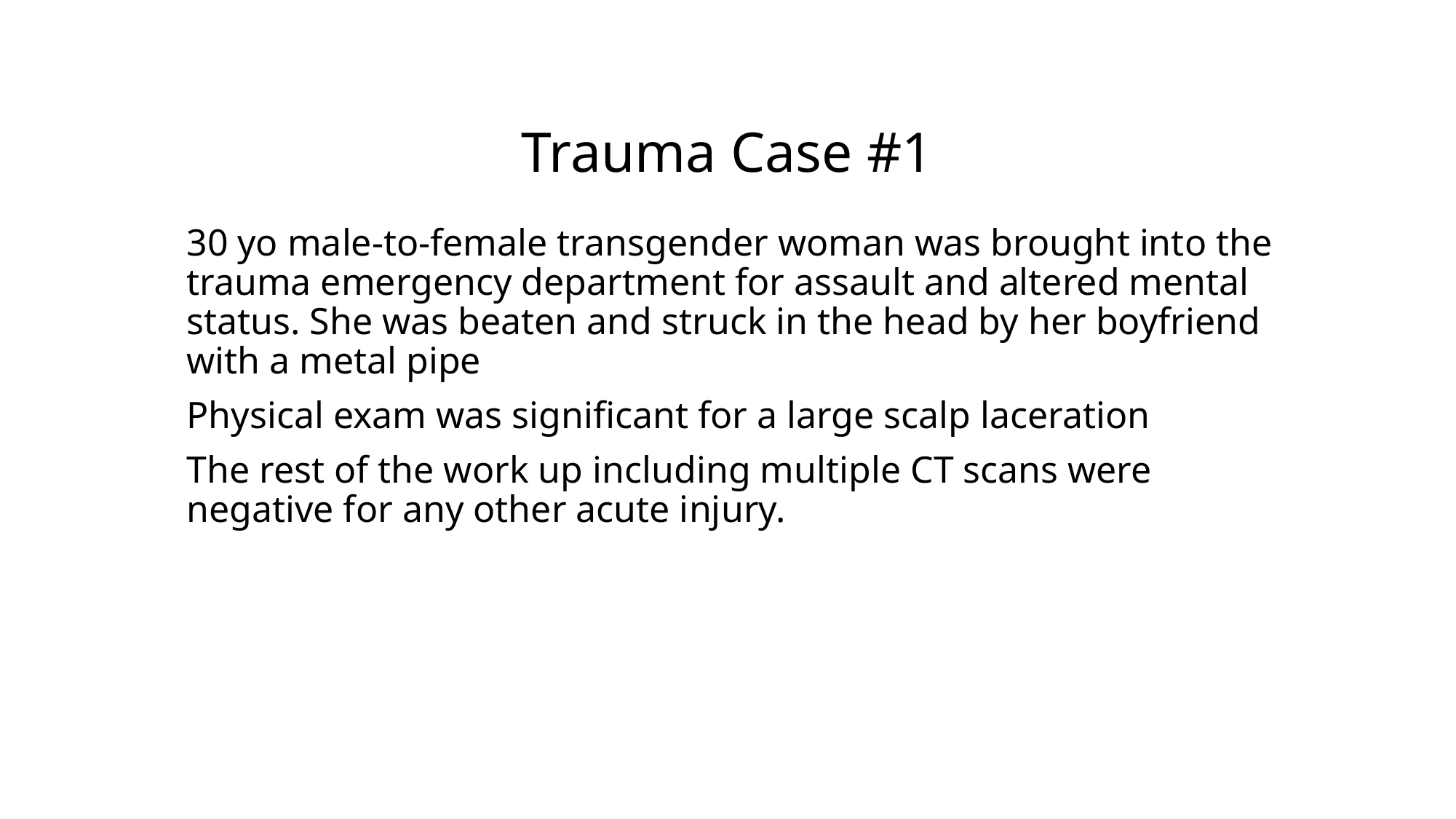

# Trauma Case #1
30 yo male-to-female transgender woman was brought into the trauma emergency department for assault and altered mental status. She was beaten and struck in the head by her boyfriend with a metal pipe
Physical exam was significant for a large scalp laceration
The rest of the work up including multiple CT scans were negative for any other acute injury.

## Slide 31
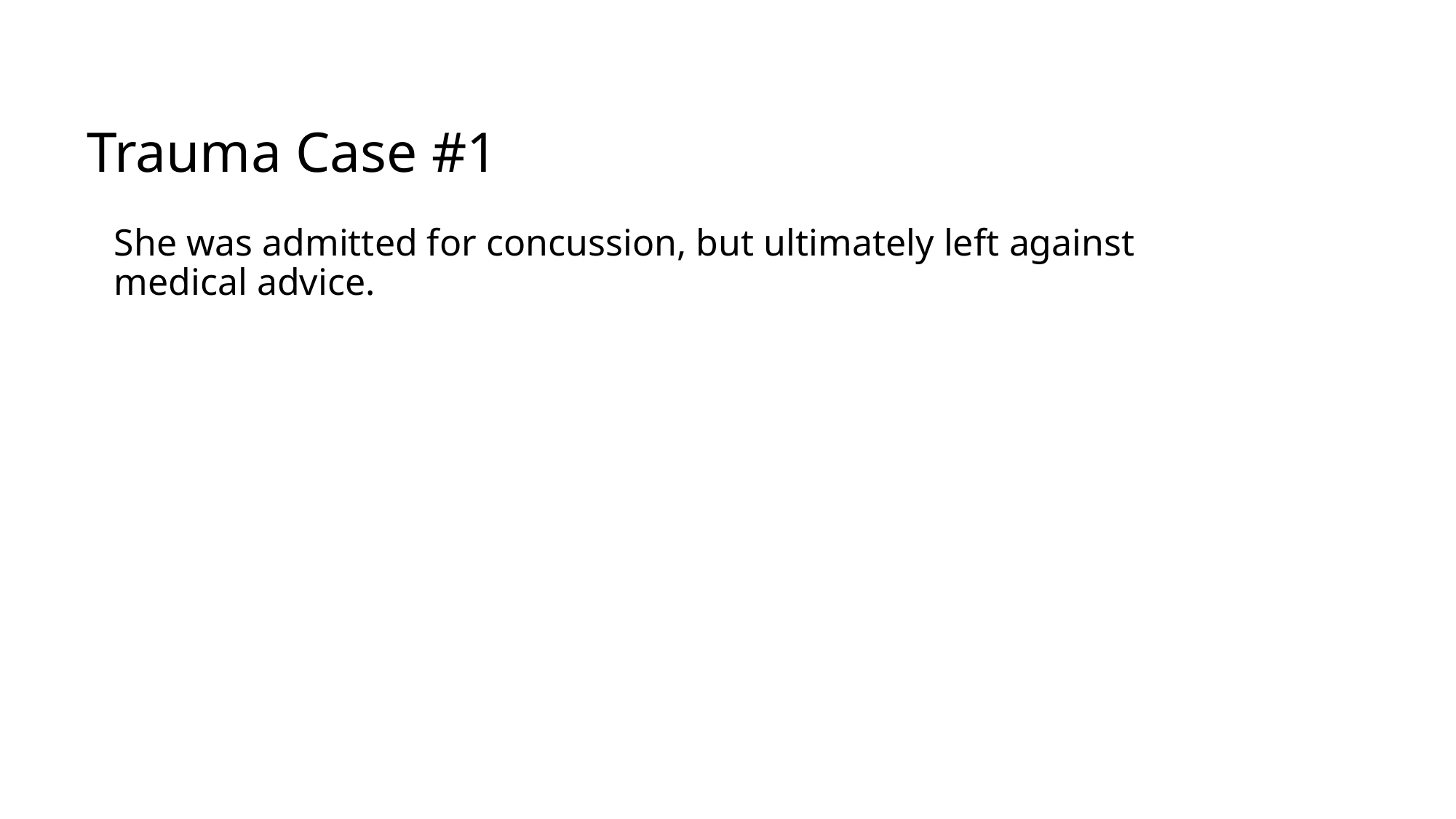

# Trauma Case #1
She was admitted for concussion, but ultimately left against medical advice.

## Slide 32
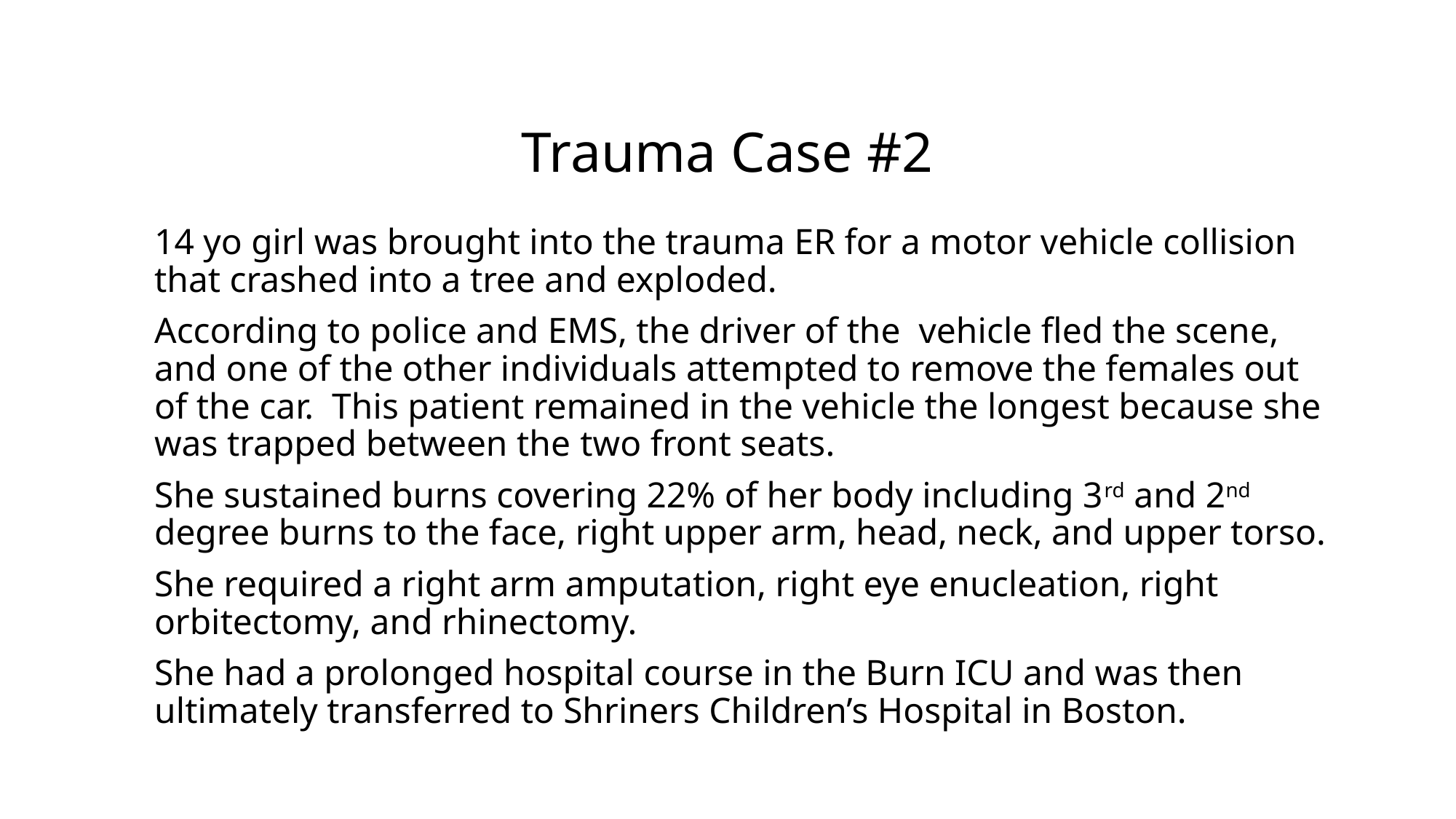

# Trauma Case #2
14 yo girl was brought into the trauma ER for a motor vehicle collision that crashed into a tree and exploded.
According to police and EMS, the driver of the vehicle fled the scene, and one of the other individuals attempted to remove the females out of the car. This patient remained in the vehicle the longest because she was trapped between the two front seats.
She sustained burns covering 22% of her body including 3rd and 2nd degree burns to the face, right upper arm, head, neck, and upper torso.
She required a right arm amputation, right eye enucleation, right orbitectomy, and rhinectomy.
She had a prolonged hospital course in the Burn ICU and was then ultimately transferred to Shriners Children’s Hospital in Boston.

## Slide 33
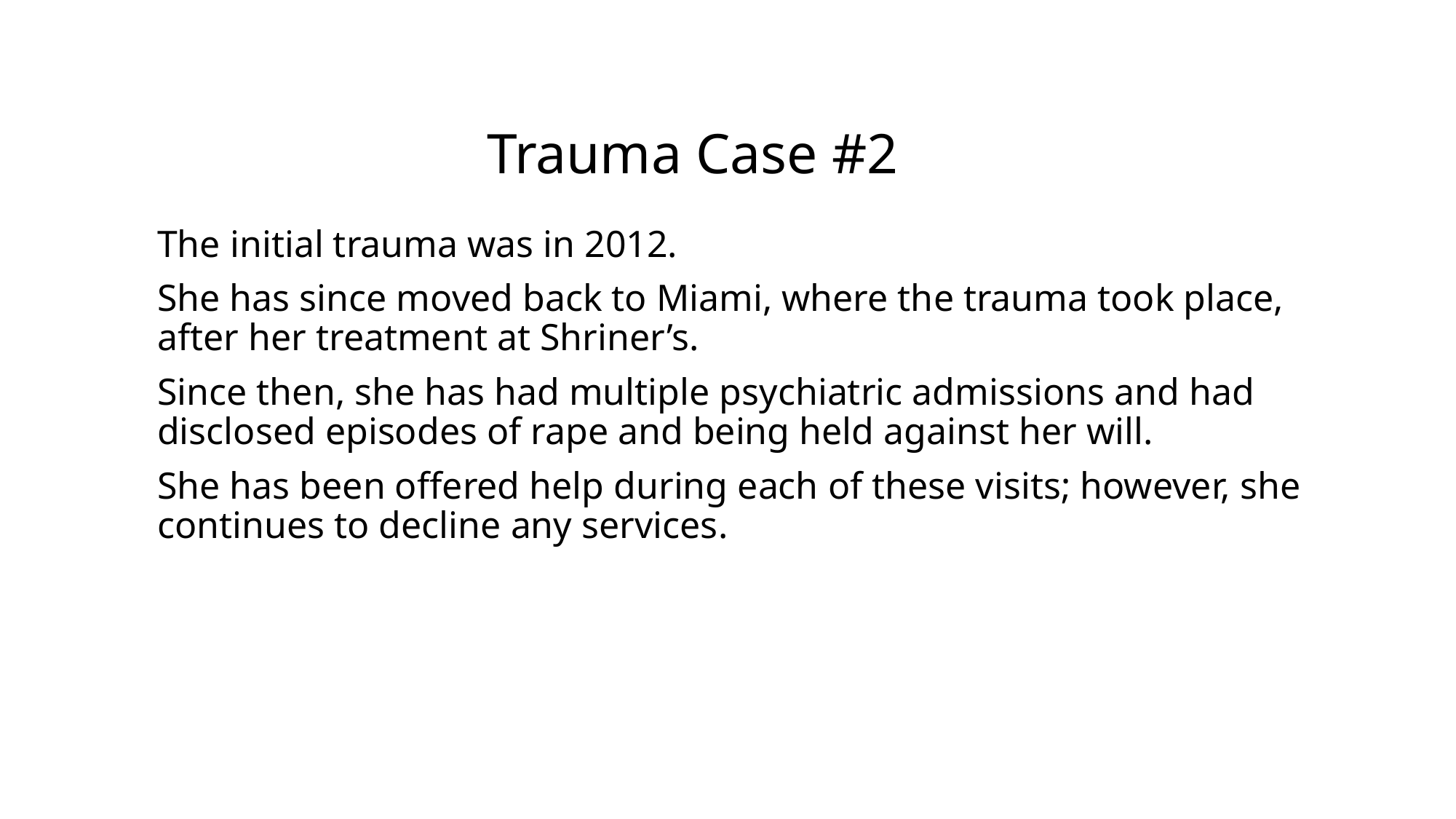

Trauma Case #2
The initial trauma was in 2012.
She has since moved back to Miami, where the trauma took place, after her treatment at Shriner’s.
Since then, she has had multiple psychiatric admissions and had disclosed episodes of rape and being held against her will.
She has been offered help during each of these visits; however, she continues to decline any services.

## Slide 34
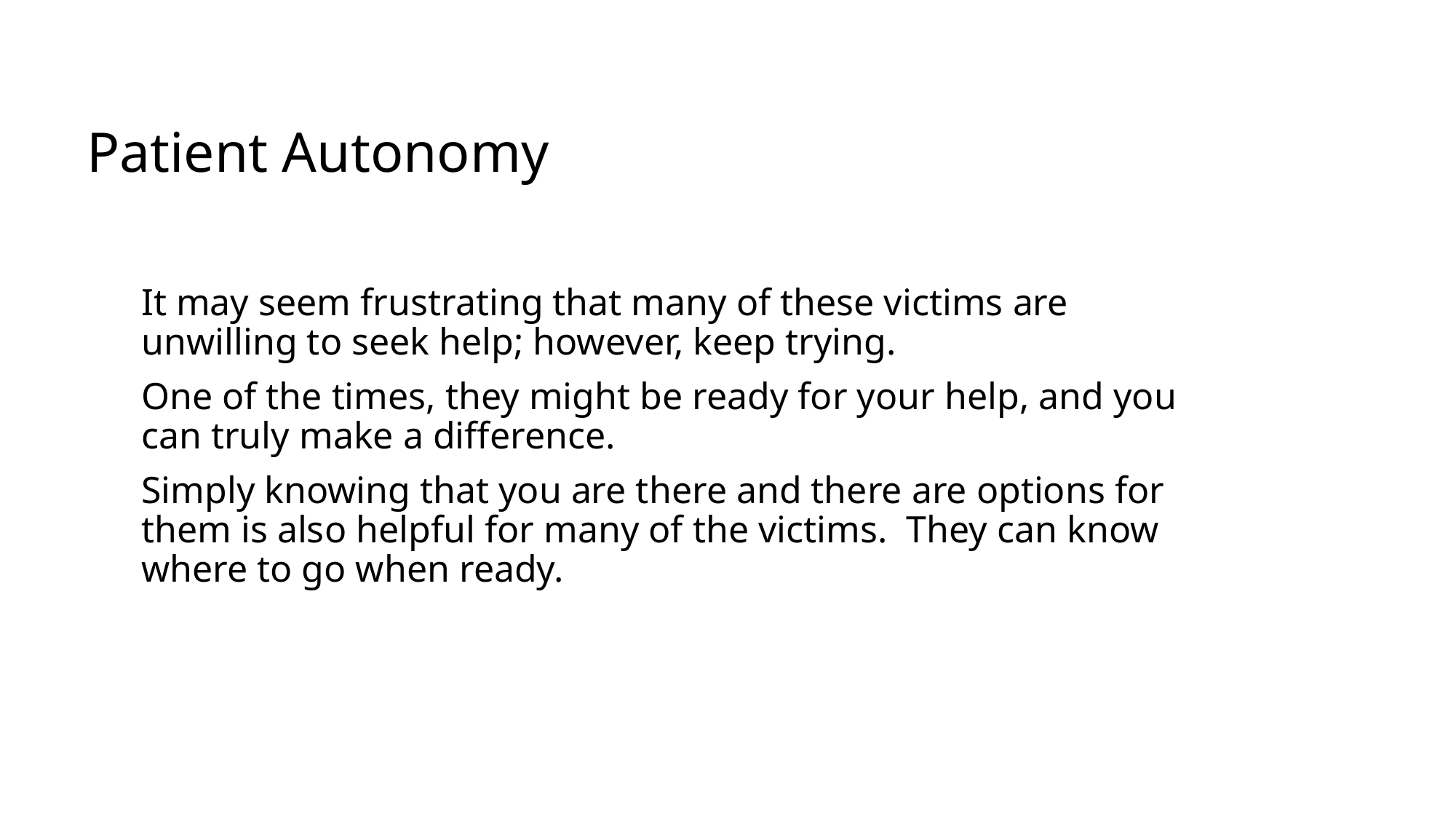

# Patient Autonomy
It may seem frustrating that many of these victims are unwilling to seek help; however, keep trying.
One of the times, they might be ready for your help, and you can truly make a difference.
Simply knowing that you are there and there are options for them is also helpful for many of the victims. They can know where to go when ready.

## Slide 35
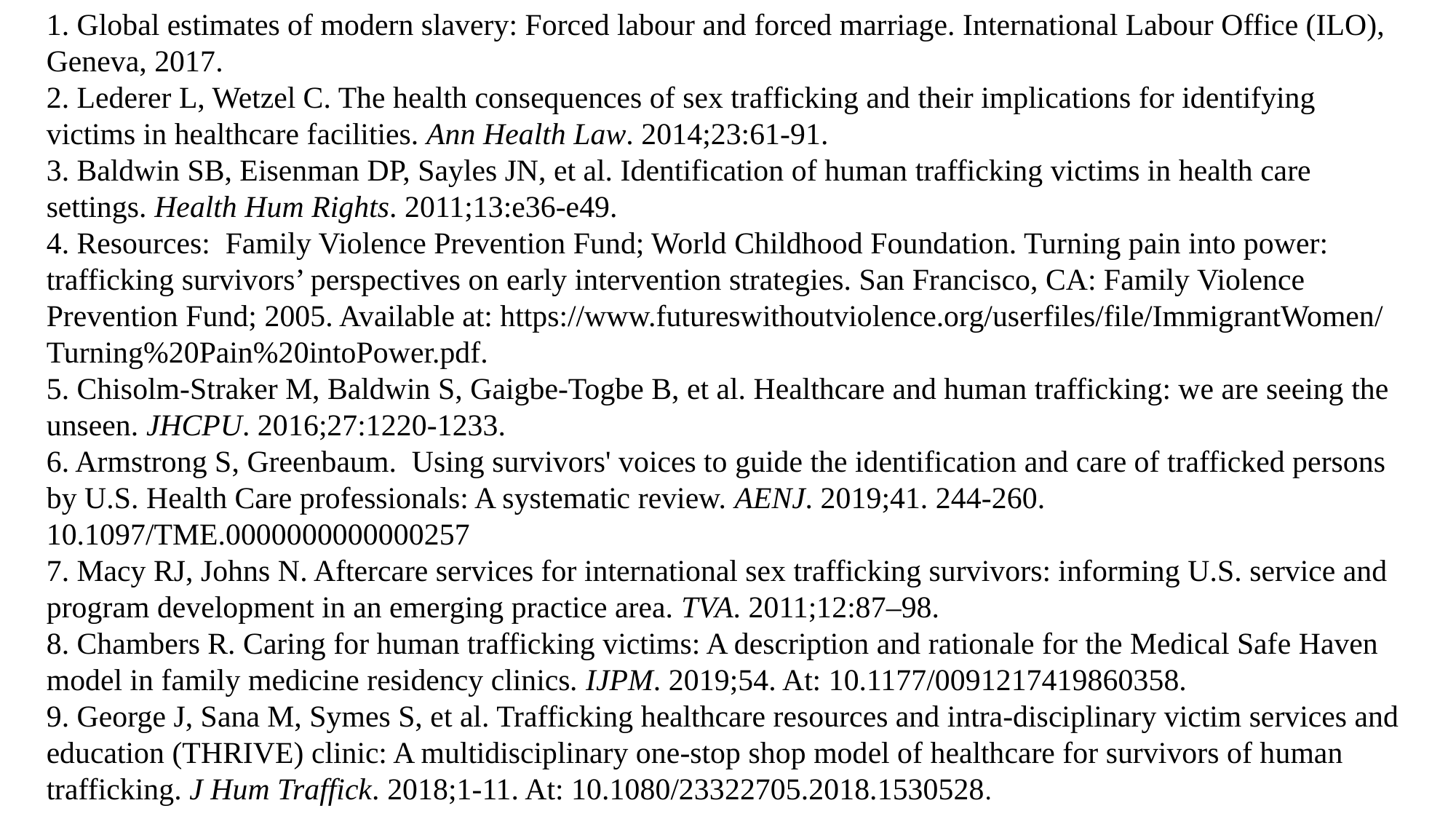

References
1. Global estimates of modern slavery: Forced labour and forced marriage. International Labour Office (ILO), Geneva, 2017.
2. Lederer L, Wetzel C. The health consequences of sex trafficking and their implications for identifying victims in healthcare facilities. Ann Health Law. 2014;23:61-91.
3. Baldwin SB, Eisenman DP, Sayles JN, et al. Identification of human trafficking victims in health care settings. Health Hum Rights. 2011;13:e36-e49.
4. Resources: Family Violence Prevention Fund; World Childhood Foundation. Turning pain into power: trafficking survivors’ perspectives on early intervention strategies. San Francisco, CA: Family Violence Prevention Fund; 2005. Available at: https://www.futureswithoutviolence.org/userfiles/file/ImmigrantWomen/Turning%20Pain%20intoPower.pdf.
5. Chisolm-Straker M, Baldwin S, Gaigbe-Togbe B, et al. Healthcare and human trafficking: we are seeing the unseen. JHCPU. 2016;27:1220-1233.
6. Armstrong S, Greenbaum. Using survivors' voices to guide the identification and care of trafficked persons by U.S. Health Care professionals: A systematic review. AENJ. 2019;41. 244-260. 10.1097/TME.0000000000000257
7. Macy RJ, Johns N. Aftercare services for international sex trafficking survivors: informing U.S. service and program development in an emerging practice area. TVA. 2011;12:87–98.
8. Chambers R. Caring for human trafficking victims: A description and rationale for the Medical Safe Haven model in family medicine residency clinics. IJPM. 2019;54. At: 10.1177/0091217419860358.
9. George J, Sana M, Symes S, et al. Trafficking healthcare resources and intra-disciplinary victim services and education (THRIVE) clinic: A multidisciplinary one-stop shop model of healthcare for survivors of human trafficking. J Hum Traffick. 2018;1-11. At: 10.1080/23322705.2018.1530528.
