# Supplementary material for: Identification of a Human Trafficking Victim: A Simulation
Source: J Educ Teach Emerg Med. 2024 Jul 31;9(3):S1–S29. doi: 10.21980/J8293F (PMC11312875; doi:10.21980/J8293F)
Supplement: Supplementary file 2 [file 9-3-S1-Supp2.pptx]

## Slide 1
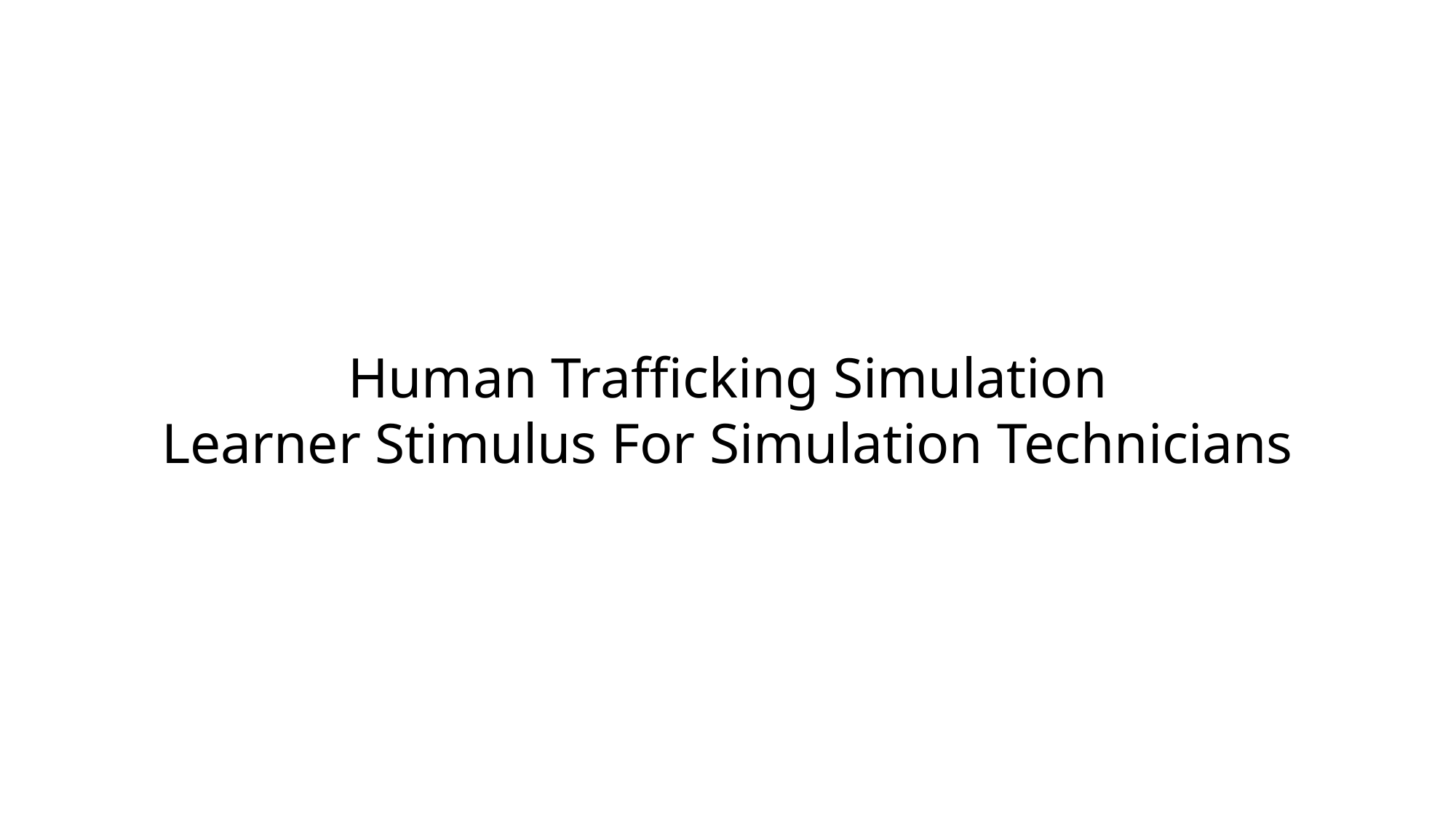

Human Trafficking Simulation
Learner Stimulus For Simulation Technicians

## Slide 2
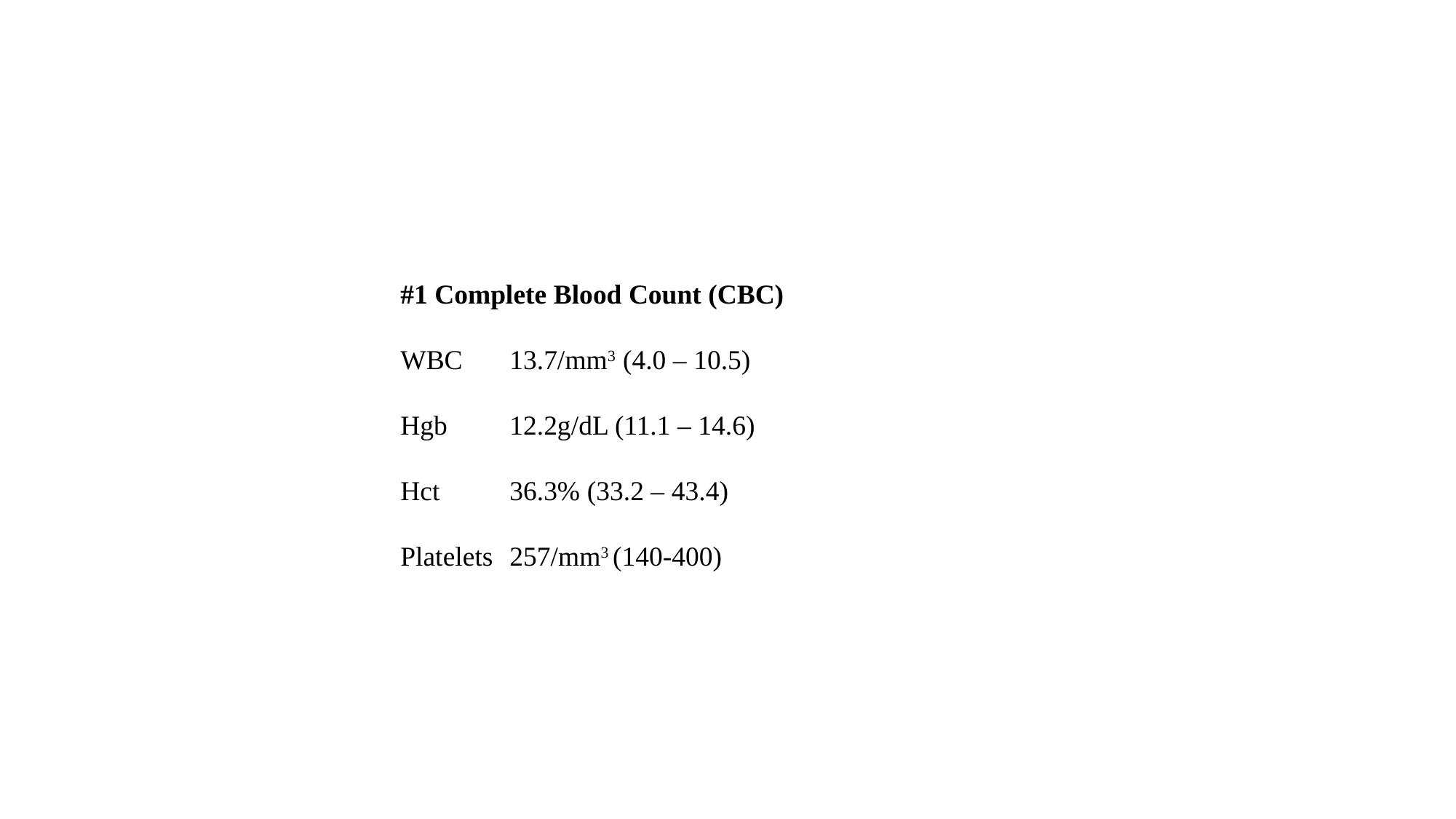

#1 Complete Blood Count (CBC)
WBC	13.7/mm3	 (4.0 – 10.5)
Hgb	12.2g/dL (11.1 – 14.6)
Hct	36.3% (33.2 – 43.4)
Platelets	257/mm3 (140-400)

## Slide 3
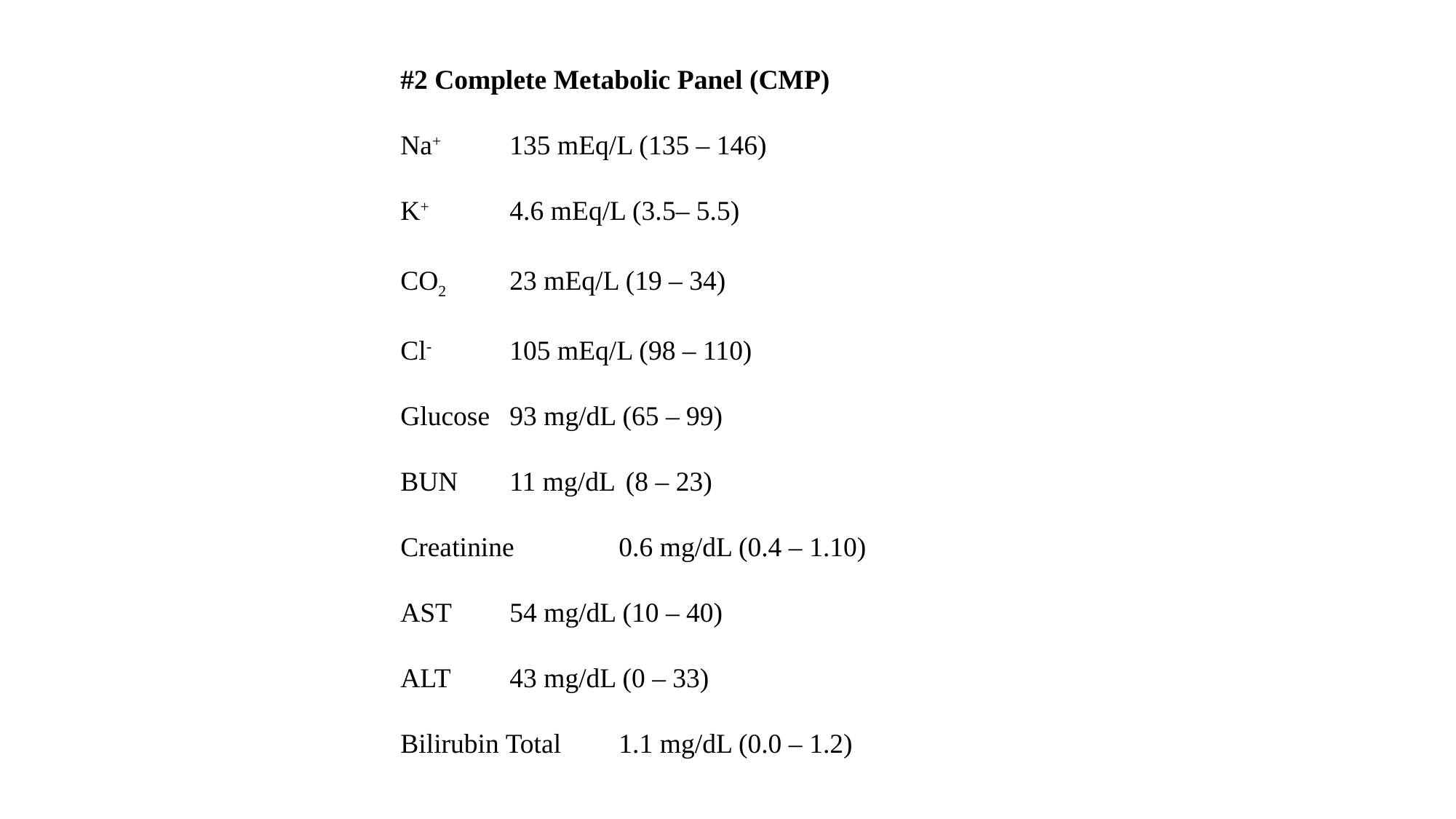

#2 Complete Metabolic Panel (CMP)
Na+ 	135 mEq/L (135 – 146)
K+ 	4.6 mEq/L (3.5– 5.5)
CO2 	23 mEq/L (19 – 34)
Cl- 	105 mEq/L (98 – 110)
Glucose 	93 mg/dL (65 – 99)
BUN 	11 mg/dL	 (8 – 23)
Creatinine 	0.6 mg/dL (0.4 – 1.10)
AST	54 mg/dL (10 – 40)
ALT	43 mg/dL (0 – 33)
Bilirubin Total	1.1 mg/dL (0.0 – 1.2)

## Slide 4
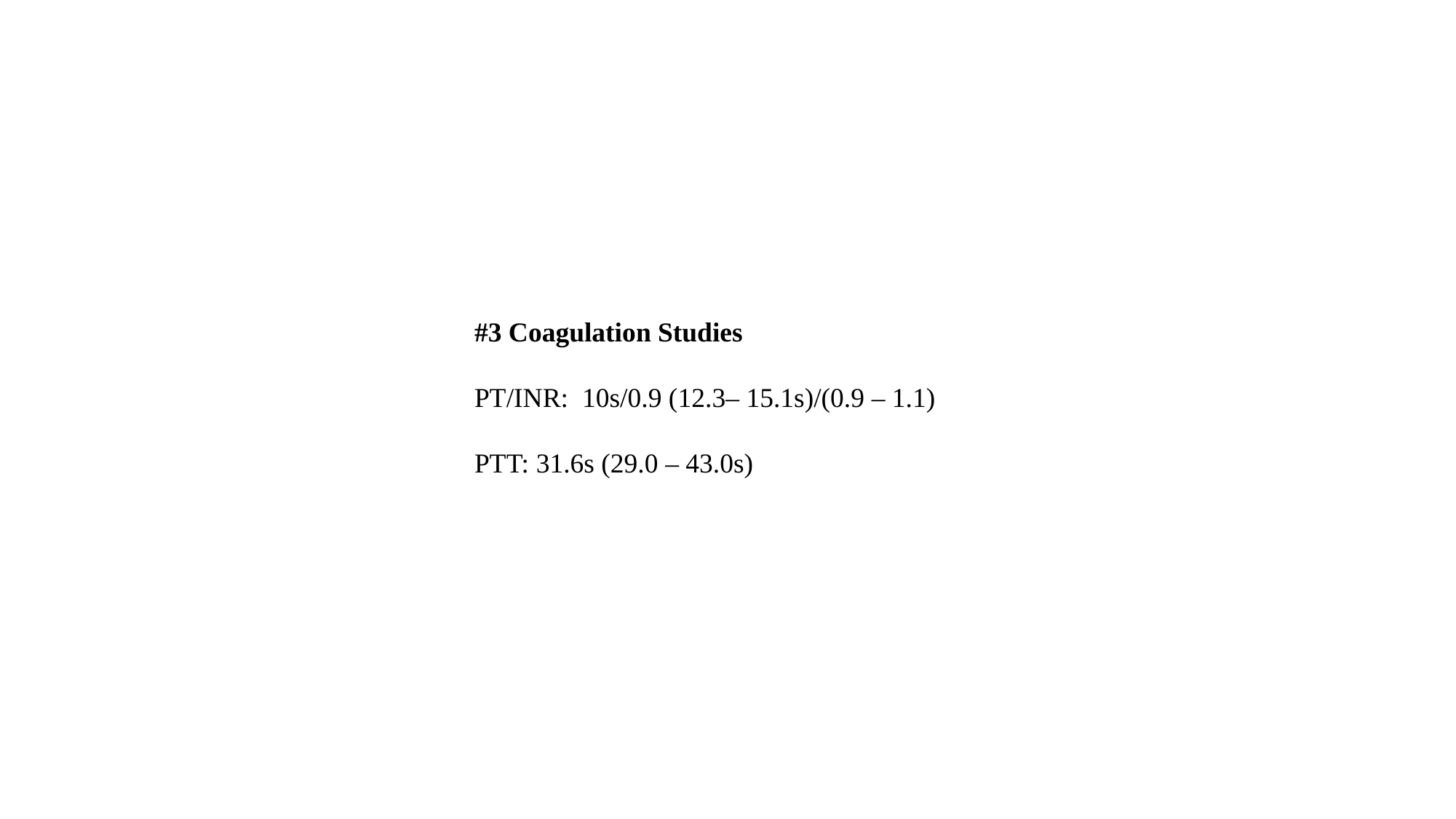

#3 Coagulation Studies
PT/INR: 10s/0.9 (12.3– 15.1s)/(0.9 – 1.1)
PTT: 31.6s (29.0 – 43.0s)

## Slide 5
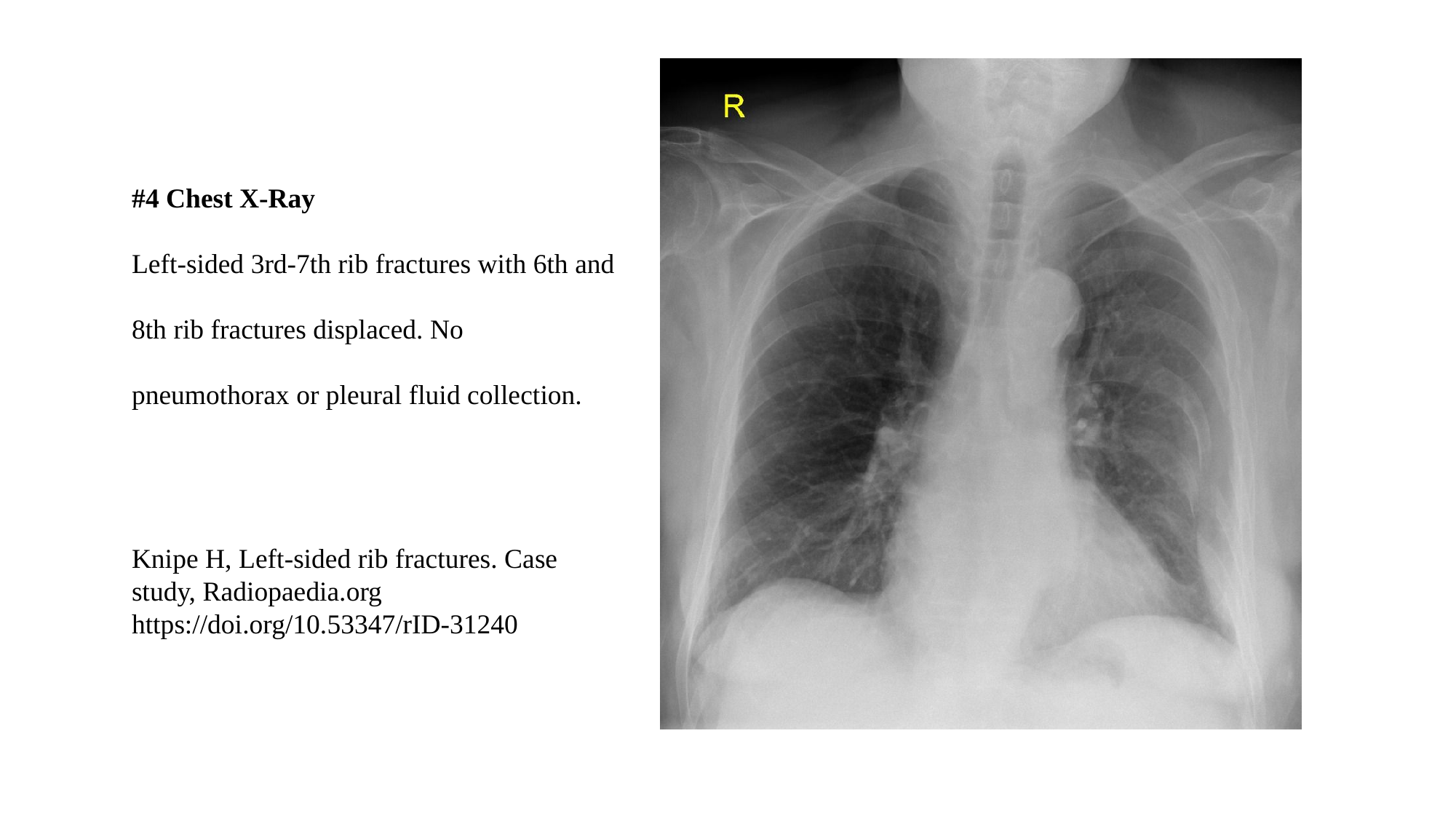

#4 Chest X-Ray
Left-sided 3rd-7th rib fractures with 6th and 8th rib fractures displaced. No pneumothorax or pleural fluid collection.
Knipe H, Left-sided rib fractures. Case study, Radiopaedia.org https://doi.org/10.53347/rID-31240

## Slide 6
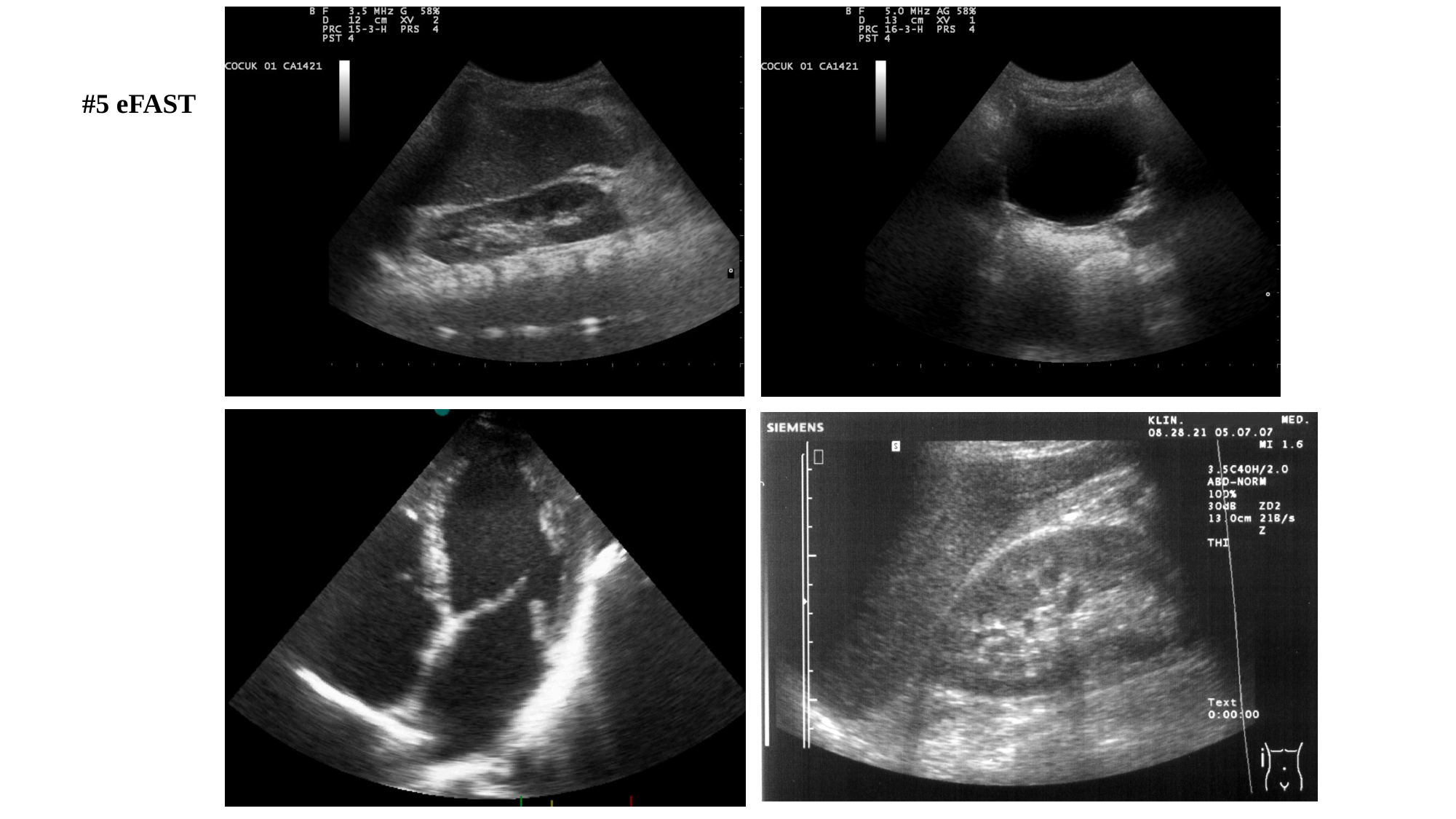

#5 eFAST

## Slide 7
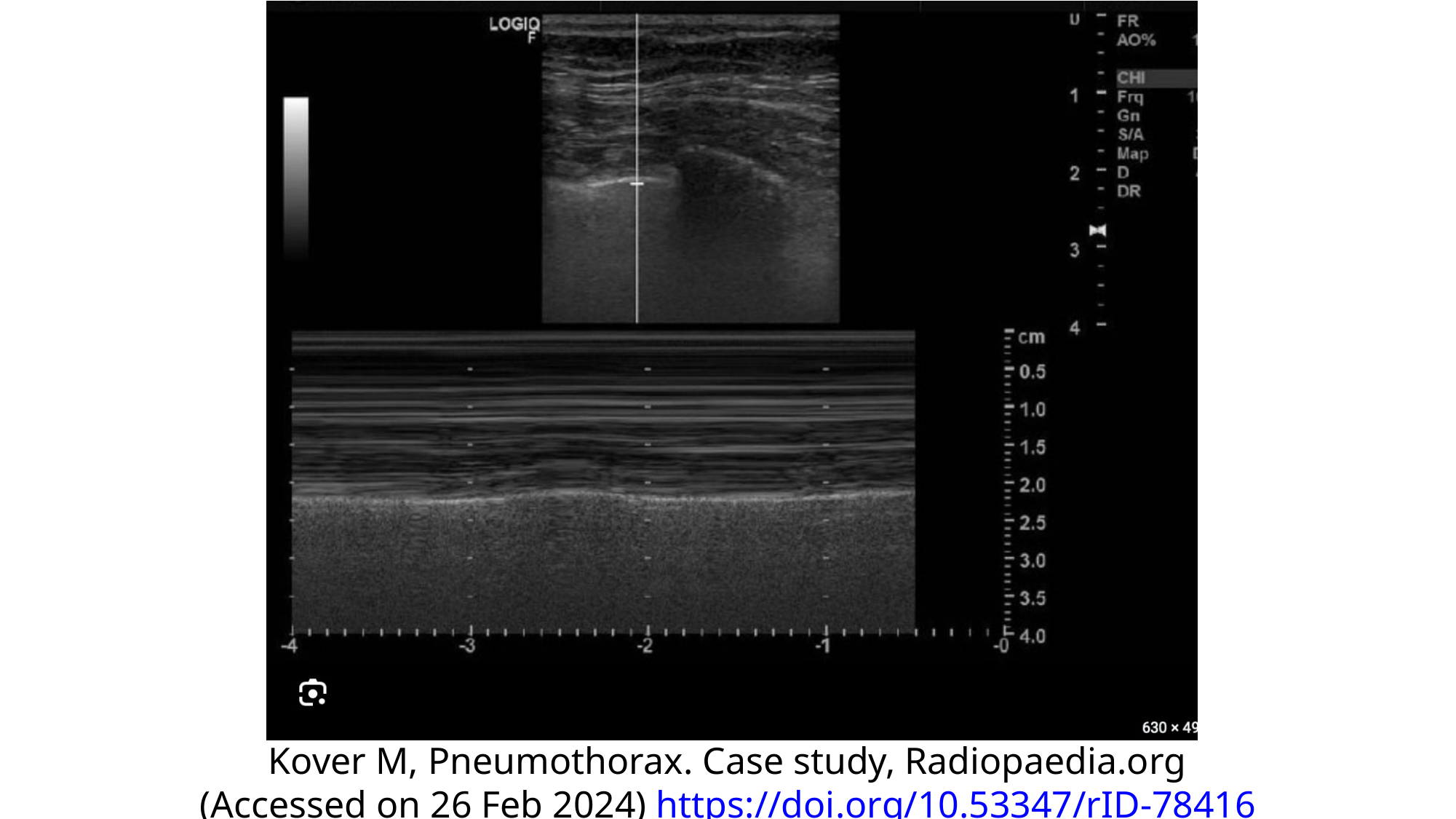

Kover M, Pneumothorax. Case study, Radiopaedia.org (Accessed on 26 Feb 2024) https://doi.org/10.53347/rID-78416

## Slide 8
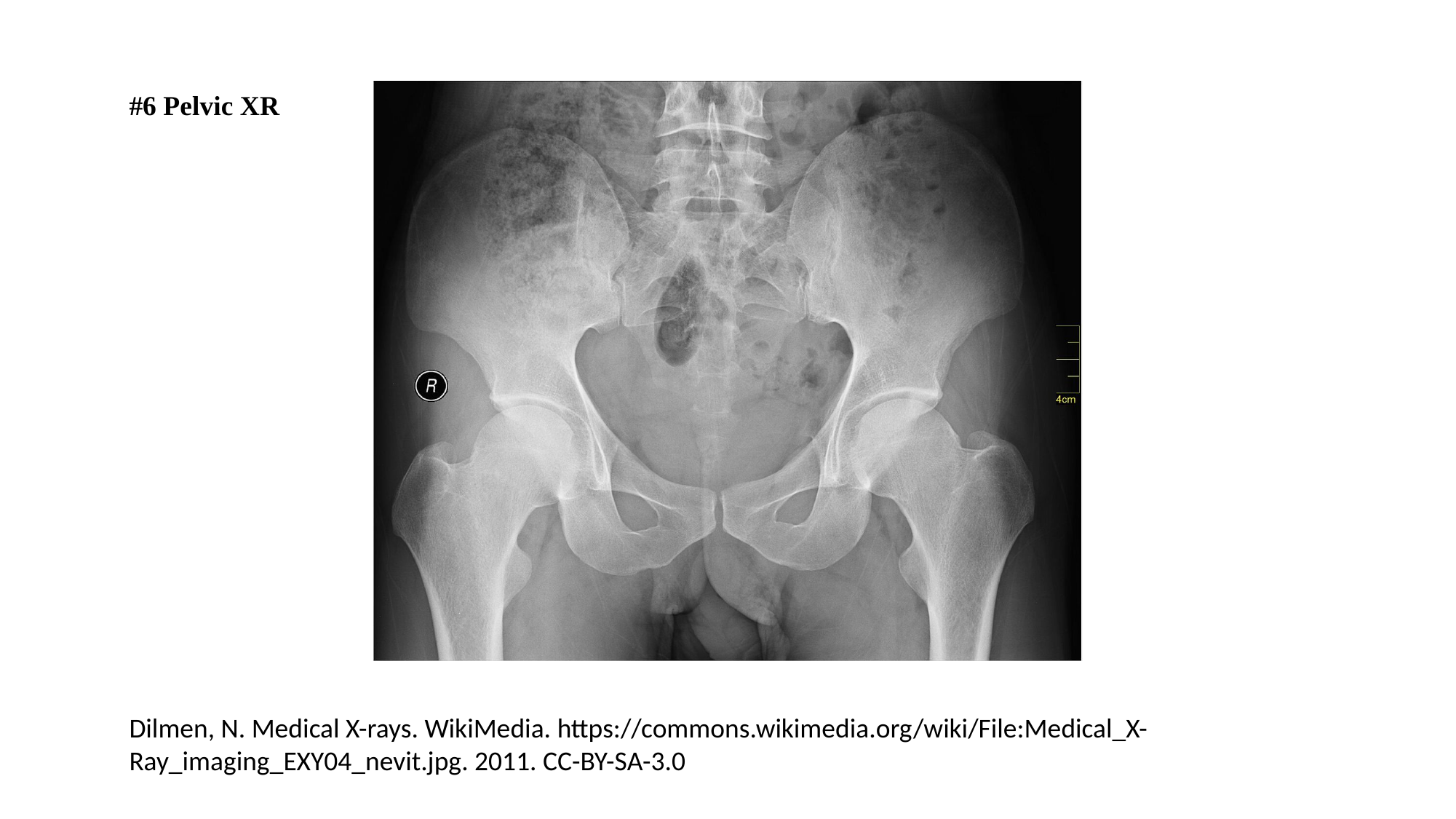

#6 Pelvic XR
Dilmen, N. Medical X-rays. WikiMedia. https://commons.wikimedia.org/wiki/File:Medical_X-Ray_imaging_EXY04_nevit.jpg. 2011. CC-BY-SA-3.0
